# Family Formation and Employment Changes Among Descendants of Immigrants in France: A Multiprocess Analysis

**DOI:** 10.1007/s10680-024-09709-3

**Published:** 2024-09-09

**Authors:** Isaure Delaporte, Hill Kulu

**Affiliations:** 1 University of St Andrews, St Andrews, UK; 2 Present Address: Research Department, International Labour Organization, Geneva, Switzerland

**Keywords:** Fertility, Employment, Life-course events, Multi-level event history analysis, Descendants of immigrants, France

## Abstract

This paper investigates the association between family formation and the labour market trajectories of immigrants’ descendants over the life course. Using rich data from the *Trajectories and Origins* survey from France, we apply multilevel event history models to analyse the transitions in and out of employment for both men and women by parity. We account for unobserved co-determinants of childbearing and employment by applying a simultaneous-equations modelling. Our analysis shows that women’s professional careers are negatively associated with childbirth. There are differences across descendant groups. The female descendants of Turkish immigrants are more likely to exit employment and less likely to re-enter employment following childbirth than women from other groups. The negative impact of childbearing on employment is slightly overestimated among women due to unobserved selection effects. Among men, the descendants of European immigrants are less likely to exit employment after having a child than other descendant groups. The study demonstrates the negative effect of childbearing on women’s employment, which is pronounced for some minority groups suggesting the need for further policies to help women reconcile work with family life.

## Introduction

European labour markets are characterised by gender disparities. Women continue to have lower labour force participation rates than men (ILO, 2018). Besides, women work fewer hours, are concentrated in specific occupations, and earn less (OECD, 2021). One important root cause of gender inequality in the labour market is family formation. A large body of literature has found that women’s professional careers are negatively affected following childbirth, whereas men’s employment trajectories are not (Aassve et al., 2006; Angelov et al., 2016; Bertrand et al., 2010; Kleven & Landais, 2017; Kleven et al., 2019a; Kreyenfeld, 2015; Loughran & Zissimopoulos, 2009; Wilner, 2016). Some groups are more affected than others: immigrant women experience a greater motherhood penalty than native women (Achouche, 2022; Kil et al., 2018; Nieto, 2021; Vidal-Coso, 2019). Still, more research is needed on the effect of childbirth on the employment trajectories of male and female descendants of immigrants.

The effect of having a child on individuals’ labour market decisions is likely to differ across population groups for a number of reasons. First, the descendants of immigrants often differ from each other and from the native population (here defined as native-born individuals with two native-born parents) in their cultural background, social norms, and preferences. They hold different preferences about the timing of family formation (Delaporte & Kulu, 2022; Kulu et al., 2021) and have different expectations regarding the division of paid and unpaid work (Fleischmann & Höhne, 2013; Khoudja & Platt, 2018). Male and female descendants of immigrants do not fare equally in the labour market (Algan et al., 2010; Di Stasio & Larsen, 2020; Petit et al., 2013; Silberman et al., 2007) and individuals who have limited labour market opportunities might be more inclined to exit the labour market following childbirth. Furthermore, the descendants of immigrants may differ from each other in the number of family members available for informal care or in their attitudes to childcare (Biegel et al., 2021; Seibel & Hedegaard, 2017). Thus, it is important to investigate potential differences in the effect of childbirth on employment by gender and migration background.

This paper investigates the relationship between family formation and the labour market trajectories of immigrants’ descendants. We focus on France which provides a rich context for the study of differences in employment trajectories among diverse population groups. The minority population comprises a number of sizeable groups, with differentiated employment histories, and family patterns (Delaporte & Kulu, 2022). We use a French survey—*Trajectories and Origins*—which holds information on immigrants, immigrants’ descendants, and French natives, and contains retrospective biographical information on individuals’ childbearing events and their labour market outcomes over the life course. We examine the employment trajectories of both men and women and focus on the descendants of immigrants (including immigrants who have arrived in France before the age of 15) who belong to six origin groups, namely the descendants of North Africans, sub-Saharan Africans, South East Asians, Turkish, Southern Europeans, and other Europeans. We apply multilevel event history models to study repeated events of employment changes as well as the birth of several children. We examine three sets of transitions: (i) the transition to first employment after leaving full-time education, (ii) the transitions out of employment, and (iii) the transitions to second- and higher-order employment. For each set of transitions, we investigate the relative risks of experiencing these changes separately for men and women by parity. We also explore differences between immigrants’ descendants and natives as well as across origin groups.

This study extends previous research in the following ways. First, although the link between fertility and employment has been investigated extensively among the majority population (Aassve et al., 2006; Angelov et al., 2016; Bertrand et al., 2010; Kleven & Landais, 2017; Kleven et al., 2019a; Kreyenfeld, 2015; Loughran & Zissimopoulos, 2009; Wilner, 2016), few studies have examined how childbearing is related to the employment pathways of immigrants (Achouche, 2022; Lacroix & Vidal-Coso, 2019; Nieto, 2021; Vidal-Coso, 2019) or those of their descendants (Kil et al., 2018; Maes et al., 2021; Samper Mejia, 2023). In this study, we analyse how family and employment interact from an intersectional perspective taking into account both gender and migration background in terms of nativity but also migrant origin. This allows us to shed light on the effect of family formation on the labour market trajectories of immigrant descendant and native men and women. We also look at differences across origin groups.

Second, we contribute to the literature on the economic integration of the descendants of immigrants (Algan et al., 2010; Clark & Drinkwater, 2010; Clark & Ochmann, 2022; Meurs et al., 2006; Piton & Rycx, 2020; Silberman et al., 2007; Zwysen & Demireva, 2020) by studying repeated events of employment changes. Most studies focus on one single transition in the entire professional career of individuals (Ganault & Pailhé, 2022). In contrast, examining all events over the life course enables us to shed light on the extent to which patterns of entry or exit explain variation in labour force participation across descendant groups. While higher rates of employment exit might indicate issues around retention or instability, lower rates of employment entry are more likely to be a signal of structural or cultural obstacles (Khoudja & Platt, 2018). Therefore, examining differences in both employment entry and exit rates will allow us to better understand some of the obstacles encountered by individuals from different groups.

Lastly, most research on fertility and employment has not accounted for possible unobserved selection effects. Yet, when studying the effect of childbirth on employment, there are potential unobserved selection effects as individuals who are more likely to change their employment status may also be more (or less) likely to have a birth because of unobserved characteristics. This would lead to a biased estimation of the effect of childbirth on employment. In this paper, we adopt a simultaneous-equations modelling approach which allows us to detect and control for unobserved time-constant co-determinants of these two processes. Although simultaneous-equations hazard models have been used in research on interrelated event histories of individuals before (Aassve et al., 2006; Kulu & Steele, 2013; Matysiak, 2009; Mikolai & Kulu, 2018; Steele et al., 2005, 2006), to the best of our knowledge, no study has applied this method to investigate the interrelationship between employment and childbearing dynamics among migrant and ethnic minority populations.

## Theoretical Framework

### Work–Family Balance: A Gendered Perspective

A large body of research highlights the role of family formation in explaining gender inequality in the labour market (Angelov et al., 2016; Bertrand et al., 2010; Kleven & Landais, 2017; Kleven et al., 2019a; Kreyenfeld, 2015; Loughran & Zissimopoulos, 2009; Wilner, 2016). While men typically maintain stable labour force participation throughout their lives, women often experience fluctuations tied to childbirth and family responsibilities (Aassve et al., 2006; Angrist & Evans, 1996; Herrarte et al., 2012; Jacobsen et al., 1999; Kleven et al., 2019a; Sieppi & Pehkonen, 2019).

The transition into motherhood often results in lower employment rates (Cristia, 2008; Fitzenberger et al., 2013; Gutierrez-Domenech, 2005a; Michaud & Tatsiramos, 2011), reduced earnings (Angelov et al., 2016; Kleven et al., 2019b, 2019c), and fewer working hours (Begall & Grunow, 2015; Gutierrez-Domenech, 2005b; Kleven et al., 2019a; Lundberg & Rose, 2000; Miller, 2011; Wood et al., 2016). Moreover, childbirth diminishes women’ performance (Azmat & Ferrer, 2017), experience (Daniel et al., 2013; Klepinger et al., 1999), occupational status (Cools et al., 2017; Kleven et al., 2019a), productivity (Krapf et al., 2017), as well as full-time (Daniel et al., 2013; Paull, 2008) and high-paid private sector employment (Daniel et al., 2013; Kleven et al., 2019a; Lundborg et al., 2017). Many women move to more family-friendly workplaces after having children (Hotz et al., 2018).

The negative effect of childbirth on women’ professional careers stems from various factors (Fiori & Di Gessa, 2022; Matysiak & Cukrowska-Torzewska, 2021). First, career interruptions and reduced working hours contribute to human capital depreciation. It may also signal lower labour market attachment to employers, hindering promotion prospects for women who opt for long parental leaves (Evertsson, 2016; Evertsson & Duvander, 2011; Puhani & Sonderhof, 2011). Second, mothers may display worse labour market outcomes because they choose jobs which are more aligned with childcare, but these positions often pay lower wages and offer limited advancement opportunities. Finally, having children may also affect mothers’ productivity and thereby subsequent labour market outcomes.

In contrast, research on fatherhood’s impact on labour market trajectories yields mixed findings. Most previous studies have shown that fathers may experience a “fatherhood premium” characterised by higher wages and occupational status compared to childless men. This is attributed to a selection of highly successful men into parenthood (Baranowska-Rataj & Matysiak, 2022), increased work effort of new fathers who see themselves as the primary breadwinner of the family, and discriminatory practices of employers who perceive fathers as highly reliable and committed employees (Hodges & Budig, 2010). Yet, recent studies have also shown that an increase in men’s involvement in the family may also affect their professional careers, leading to wage penalties for taking parental leave or using flexible work arrangements, penalties that can even be higher than those experienced by mothers (Evertsson, 2016; Rudman & Mescher, 2013). Other studies find that the effect differs across men’s wage distribution, and that there are premia among higher earning men (Cooke, 2014; Glauber, 2018) and penalties for low earning ones (Cooke, 2014). Therefore, more research is needed to understand the effect of family formation on men’ employment trajectories.

### Work–Family Balance Among Migrant Populations

An interesting subsequent question is to which extent the effect of childbirth on labour market dynamics differ across population groups. A number of studies have examined the influence of childbirth on the employment pathways of immigrants. For instance, Lacroix and Vidal-Coso (2019) find a greater drop in the probability of being employed for immigrant women in more affluent households compared to native women. More recently, a number of studies have adopted a longitudinal approach; they show that employment levels decrease to a larger extent following the transition to parenthood among migrant women than among natives (Kil et al., 2018; Nieto, 2021; Vidal-Coso, 2019). The negative effect of motherhood on wages is also found to be higher for immigrants than it is for the native population (Achouche, 2022). There are substantial differences across migrant origin groups. In France, Achouche (2022) asserts the existence of an especially pronounced motherhood penalty for mothers from the Maghreb (considering the impact of childbirth on wages).

Few studies examine how childbearing relates to the employment pathways of the descendants of immigrants compared to those of natives (Maes et al., 2021; Milewski & Adserà, 2023; Samper Meja, 2023). Findings differ according to the national context: Holland and de Valk (2017) find that the gap in labour force participation between mothers and childless women was similar for native and second-generation Turkish women in Germany and Sweden but was larger in the Netherlands and France. There are also differences across origin groups. In Germany, Samper Meja (2023) finds that trajectories of low labour market participation are highly related to trajectories of early family formation and that compared to other origin groups, the female descendants of Turkish immigrants were more likely to be on an early family formation path, and a path with multiple non-employment spells. In the UK, Khoudja and Platt (2018) find that Pakistani and Bangladeshi women’s labour market entries and exits are less sensitive to childbearing events compared to those of other women.

### The Intersectionality of Gender and Migration Background

The implications of the intersection of multiple social identities have rarely been considered in the work–family literature (Hwang & Hoque, 2023). Yet, the relationship between employment and childbearing might vary by gender and migration background for a number of reasons.

First, there are large strands of literature indicating that the descendants of immigrants differ from each other and from the native population in their social norms, and preferences. This is frequently discussed in the context of the socialisation and adaptation hypotheses. For migrant populations, the *socialization hypothesis* considers that the values and norms individuals have been exposed to in their upbringing influence their decisions and behaviours over the life course while the *adaptation hypothesis* considers the living and opportunity costs associated with the host country but also its cultural norms the most important (Kulu, 2005). For the descendants of immigrants, growing up in the host country makes them more likely to be under the influence of the *mainstream society*, leading to the adoption of host cultural values. This would indicate that they tend to endorse traditional family values of interdependence to a lesser extent than their parents. On the other hand, they may also grow up under the influence of a *minority subculture* and thus family values of interdependence may resist acculturation and persist in the second generation (Idema & Phalet, 2007). In practice, the descendants of immigrants often exhibit multiple identities (Delaporte, 2019; Sabatier, 2008), and this influences their decisions over the life course. Berry (1997), for instance, identified four mutually exclusive acculturation strategies: assimilation, integration, separation, and marginalisation. Several factors contribute to shaping an individual’s identity such as social networks, school socialisation, and experience of discrimination. These four strategies often lead to distinct economic outcomes.

Previous research has found, for instance, that the descendants of immigrants often exhibit different partnership and fertility patterns depending on their parents’ country of origin. They also hold different preferences about the timing of family formation (Delaporte & Kulu, 2022; Kulu et al., 2021). For instance, in Europe, the descendants of immigrants from culturally similar countries such as European and Western countries often have similar partnership patterns as the ones of natives (Andersson et al., 2015; Ferrari & Pailhé, 2017; Hannemann & Kulu, 2015; Hannemann et al., 2020; Pailhé, 2015). Their fertility levels are also closer to levels observed for the native population. By contrast, immigrants’ descendants from countries with conservative patterns of family formation exhibit higher marriage, lower cohabitation, and lower separation rates than the natives (Andersson et al., 2015; Delaporte & Kulu, 2022; Kuhnt & Krapf, 2020; Kulu & Hannemann, 2016a; Liu & Kulu, 2023; Mikolai & Kulu, 2022; Pailhé, 2015, 2017). Besides, their fertility levels tend to be higher than those of natives (González-Ferrer et al., 2017; Krapf & Wolf, 2016; Kulu & Hannemann, 2016b; Milewski, 2007; Van Landschoot et al., 2017).

These differences in partnership and fertility patterns could be indicative of different social and gender norms (Diehl et al., 2009; Milewski & Adserà, 2023; Röder & Mühlau, 2014), which in turn might influence differently individuals’ labour supply following childbirth. In the case of female descendants of immigrants, cultural norms may have important implications on their labour market behaviour post childbirth. A closely related strand of literature employs the epidemiological approach to examine how culture influences variations in economic outcomes. The studies confirm that differences in immigrant women’s labour force participation can, in part, be attributed to variations in female workforce engagement rates across their countries of origin, which serve as proxies for the prevailing attitudes and beliefs regarding women’s employment in those countries of origin (Antecol, 2000; Blau et al., 2011; Bredtmann & Otten, 2023; Fernández & Fogli, 2009). Similarly, differences in the number and timing of births among descendants of immigrants can be explained by norms surrounding fertility in their parents’ countries of origin (Blau et al., 2013; Chabé-Ferret, 2019; Fernández & Fogli, 2009).

In addition to differences between immigrants’ descendants and natives, gender-role theory argues that men and women encounter different expectations regarding their societal roles, with men facing greater pressure to focus on the job domain, and women on the family domain. Yet, culture, gender norms, and motherhood ideology vary across countries of origin and geo-cultural backgrounds (Diehl et al., 2009; Glas, 2023; Idema & Phalet, 2007; Inglehart & Norris, 2003; Khoudja & Fleischmann, 2017; Moriconi & Rodríguez-Planas, 2021; Sanchez Guerrero & Schober, 2021). For instance, in the United States, African American mothers exhibit work and motherhood practices that could be considered not conservative as they have declared in interviews that they should work outside of the home, be financially self-reliant, and use kin and community members as child caregivers (Dow, 2016). In comparison, gender-role norms and attitudes of women from the Maghreb are likely to be different, especially since they come primarily from highly traditional and religious Muslim countries (Diehl et al., 2009; Röder & Mühlau, 2014). A number of studies examine within-group variation in work–family balance (Hwang & Hoque, 2023). For instance, Hwang and Hoque (2023) find in Britain that daily family interference with work is associated with higher job-related guilt and subsequently lower job satisfaction. Yet, interestingly, this relationship is stronger for women than men in general, but also stronger for South Asian women than white British women (and men), and for South Asian men than white British men.

Strong religious commitment has also been found to be associated with the preservation of traditional gender-role attitudes (Diehl et al., 2009; Glas, 2023; Idema & Phalet, 2007). When investigating how gender-role values of the second generation are shaped by intergenerational as well as intercultural relations, Idema and Phalet (2007) find in Germany that second-generation daughters showed a significant shift towards more egalitarian values, but sons remained as conservative as their fathers. Lastly, intergenerational stability in gender beliefs is stronger among specific groups of immigrants. For instance, Turkish and Moroccan male immigrants in the Netherlands and Turkish immigrants in Germany have been found to report more traditional work–family plans and gender beliefs than other groups of immigrants (Sanchez Guerrero & Schober, 2021).

Beyond cultural norms and preferences, another explanation to potential differences in the association between childbearing and employment across population groups is linked to the fact that the descendants of immigrants from different origin groups do not fare equally in the labour market. A large strand of literature documents the disadvantaged labour market positions of some groups among the descendants of immigrants in Europe (Clark & Drinkwater, 2010; Clark & Ochmann, 2022; Meurs et al., 2006; Piton & Rycx, 2020; Silberman et al., 2007; Zwysen & Demireva, 2020). Algan et al. (2010) show that the labour market performance of immigrants’ descendants is worse compared to the natives in France, Germany, and the UK. Silberman et al. (2007) find in France that groups who come from former French colonies and/or are dominated by Muslims are substantially, if not severely, disadvantaged in the process of labour market entry. Furthermore, past unemployment increases the probability of current unemployment (absolute effect) particularly among second-generation Middle-Eastern, Turkish, and Southern European immigrants (Aradhya et al., 2023).

A great deal of these labour market disparities is due to labour market discrimination (Brinbaum & Guégnard, 2012; Brinbaum et al., 2015, 2018; Duguet et al., 2009; Meurs et al., 2006; Petit et al., 2013; Silberman & Fournier, 1999; Silberman et al., 2007) but also to the lack of social ties (Brinbaum, 2022). Furthermore, although levels of discrimination vary across national contexts and labour market segments, the general picture is one of severe disadvantage (Bertrand & Duflo, 2017; Di Stasio et al., 2021; Lippens et al., 2023; Quillian & Midtbøen, 2021; Quillian et al., 2019). Di Stasio et al. (2021) report alarming levels of discrimination, especially towards male applicants from more visible groups. Anti-Muslim discrimination and origin-based discrimination independently contribute to the severe disadvantage faced by ethnic and religious minorities. In particular, in France, candidates who signalled their affiliation to Islam in the job application were at a severe disadvantage compared to equally qualified candidates of different religions (Adida et al., 2010; Pierné, 2013; Valfort, 2020).

There are also substantial gender disparities in employment and discrimination (Di Stasio & Larsen, 2020; Petit et al., 2013). For instance, drawing on a field experiment conducted in five European countries, Di Stasio and Larsen (2020) find that employers prefer hiring white women over men for female-typed jobs. By contrast, women of colour do not have any advantage over men of the same race. Black and Middle-Eastern men encounter the strongest racial discrimination in male-typed jobs. By contrast, Petit et al. (2013) find that women of Asian origin in Ile-de-France have a greater probability of being invited to a job interview than other women and a greater probability of accessing employment than men of Asian origin. Therefore, the fact that access to employment varies across gender and migration background could lead to differences in labour supply patterns following childbirth as those that have limited access to employment may have fewer incentives to continue to work after a child is born. Furthermore, there is a strong path dependency of employment trajectories around parenthood for migrant women and natives (Maes et al., 2021) and the fact that female descendants of immigrants generally have a lower pre-birth labour market attachment than native women plays a role in explaining the observed migrant-native differentials in maternal employment.

Lastly, access to family policies such as formal childcare and parental leave that help to reduce work–family conflict may vary across groups (Eremenko & Unterreiner, 2023; Mussino et al., 2023). Previous research has found significant disadvantages among migrant groups in eligibility for family policy measures, and consequently in their use and impacts on further life-course patterns, compared to majority populations (Mussino & Duvander, 2023; Mussino et al., 2023). The uptake of (in)formal childcare has been found to be substantially lower among immigrants—especially non-European migrants—compared to the native population (Biegel et al., 2021; Schober & Spiess, 2013; Wall & Jose, 2004) and this constitutes an important source of the employment gap between immigrant and native mothers (Guirola & Sánchez-Domínguez, 2022). In Spain, Sánchez-Domínguez and Abenza (2021) find that the country’s employment and care regime forces female migrants to deal with their care responsibilities differently than their native counterparts. Regarding parental leave, Mussino and Duvander (2023) find in Sweden that there are great differences in uptake in relation to country of birth and reason for residence permit.

With respect to the descendants of immigrants, while second-generation individuals are born and raised in the host country of their parents, and thus, have no inherent reason to differ from the natives in terms of access to family policies, there are still cultural differences across regions regarding child upbringing that may lead to variations in the use of care (Eremenko & Unterreiner, 2023; Guirola & Sánchez-Domínguez, 2022; Leitner, 2003; Saraceno & Keck, 2010). For instance, parents with a migration background may particularly value childcare providers who share their cultural background and/or language, thus opting for informal childcare arrangements (Karoly & Gonzalez, 2011). Cultural preferences for parental care at home may also be transmitted across generations. Second-generation individuals from non-Western backgrounds may also rely more on family-based childcare, as traditional family structures often emphasise greater family support. Therefore, these potential differences in the uptake of childcare might lead to some heterogeneity in the effect of childbirth on individuals’ employment trajectories.

## Host Country Characteristics: The French Context

Potential differences across origin groups and genders are of course linked to the composition of the studied population. The focus on the French context is enlightening for several reasons. First, France is characterised by a long history of immigration and has a large immigrant population (12%: OECD/European Union, 2018). In the years following World War II, immigration came primarily from former French colonies such as Vietnam, Algeria, as well as sub-Saharan African and other Asian countries (Algan et al., 2010). Yet, later, the formation of the European Union in 1993 and its further expansion in 2004 led to massive migration movements within EU borders. Today, the most common countries of birth are Algeria, Morocco, Portugal, Tunisia, Italy, Turkey, and Spain. As a result, an increasing number of children in France grow up in immigrant families (Lê et al., 2022).

Second, these various origin groups have differentiated employment histories and family patterns (Delaporte & Kulu, 2022; Hannemann et al., 2020; Pailhé, 2015, 2017). For instance, in France, descendants of immigrants of European origin often have similar partnership patterns as the ones of natives (Pailhé, 2015). Their fertility levels are also closer to levels observed for the native population. By contrast, descendants of immigrants of Turkish and North African origin exhibit more conservative patterns of family formation than the natives, e.g. they typically display higher marriage, lower cohabitation, and lower separation rates than the natives (Delaporte & Kulu, 2022; Hannemann et al., 2020; Pailhé, 2015, 2017). Besides, their fertility levels tend to be higher than those of natives. These groups also experience divergent paces of integration into the French labour market. Immigrants’ descendants of sub-Saharan African, North African, and Turkish origin are particularly at risk of experiencing labour market discrimination (Meurs et al., 2006; Silberman et al., 2007). Moreover, levels of discrimination vary across genders in France (Di Stasio & Larsen, 2020; Petit et al., 2013).

Lastly, in France, family policies are designed to support the traditional family structure and encourage fertility rates. These policies typically include financial incentives such as family allowances, tax breaks for dependent children, and subsidised childcare services. As a result, family policies are considered to be quite generous. There are free day-care facilities open throughout the day, accommodating children as young as 3 months old (Cukrowska-Torzewska, 2017; Lucifora et al., 2017). Maternity and paternity leave provisions are also considered to be quite comprehensive, providing parents with time off work to care for newborns while maintaining job security. Consequently, research suggests that the motherhood penalty is notably less pronounced in France (Budig et al., 2012, 2016), especially for mothers with one or two children, with any penalty typically appearing after the birth of a third child. The French society also generally embraces the integration of paid work and family life. However, it is important to note that immigrants and their descendants may face distinct challenges in juggling work and family responsibilities.

## Hypotheses

In light of previous findings and characteristics of the French context, we develop the following hypotheses. First, we expect women’s professional careers to be negatively impacted by childbirth contrary to men’ careers for whom we do not except to find any significant effect (Hypothesis 1). This negative effect for women might be illustrated by lower rates of employment entry and higher rates of employment exit for women with children compared to those without children (Hypothesis 1a). We also expect this negative effect to increase as parity increases (Hypothesis 1b). Second, we expect the negative effect among women to be stronger for the descendants of immigrants than for native women (Hypothesis 2). This is due to the composition of the second-generation immigrant population in France. Indeed, most descendants of immigrants come from countries characterised by higher levels of traditionalism with respect to gender norms compared to France.

With regard to differences across origin groups, we expect this negative effect to be more pronounced among the descendants of immigrants who come from origin countries with more conservative norms, e.g. children of immigrants from North Africa, and Turkey, compared to the descendants of immigrants who come from origin countries that hold more progressive gender norms such as the children of European immigrants (Hypothesis 3). Lastly, we also expect to find a statistically significant correlation between the unobserved heterogeneity terms which would indicate the presence of unobserved time-constant co-determinants of childbearing and employment (Hypothesis 4). More specifically, we expect the negative effect of childbearing on employment to be reduced but to remain significant.

## Data and Methodology

The analysis is based on data from a rich French survey named *Trajectories and Origins* which was conducted by the French Institute for Demographic Studies and the French National Institute of Statistics and Economic Studies between September 2008 and February 2009. It contains information on immigrants, immigrants’ descendants, and French natives. Immigrant populations were oversampled to allow for the study of specific groups which would be difficult to observe otherwise.

For the purpose of this study, we focus on the descendants of immigrants (including immigrants who arrived to France before the age of 15) and French natives. We examine both men and women without imposing any restrictions on age and study period. To define individuals as immigrants’ descendants or natives, we use information on individuals’ own and their parents’ country of birth to determine their migration background. Natives are defined as individuals who were born in France with two French-born parents. Immigrants’ descendants were born in France with at least one of their parents born abroad. Lastly, immigrants who migrated to France as children younger than 15 are included in the same analyses as the second generation. The final sample is composed of 10,886 immigrants’ descendants (including 2,365 immigrants who arrived to France before the age of 15) and 3,462 natives.

The survey contains retrospective biographical data with information on the employment and childbearing histories of individuals. More specifically, we have information on the month and the year of each childbirth. We decide to subtract 7 months from the time of birth since at this time, the majority of women are aware that they are pregnant, and this knowledge may influence their subsequent employment trajectory and the one of their partners.^1^ We also have yearly information on the employment status of individuals across their life course. We convert the employment histories to a monthly format by assuming that each event happens at the end of each year. To ensure that our results are robust, we also conducted additional analyses (not reported in this study) where months were assigned randomly to each employment event. We find similar results in both analyses. Lastly, the survey also contains detailed information on individuals’ socio-demographic characteristics such as gender, birth cohort, origin group, and educational level.

To study changes in the employment status of individuals across their life courses, we estimate multilevel event history models. These models are an extension of conventional event history models: rather than analysing a single employment transition, individuals can move among different states. Regarding their employment status, individuals can either be salaried, self-employed, in education, unemployed, housewife, or other. To ensure a reasonable number of events in each category, we regroup salaried and self-employed individuals under the category “employed”. Individuals who are “out of employment” are either “unemployed”, “housewife”, or “other”. It is important to note also that, in our framework, maternity/paternity leaves (which typically last 16 and 6 weeks, respectively, in France) are not counted as employment exits.

We start by observing all individuals from the time they leave full-time education. At this point, they can move to their first employment. For all individuals, once in employment, they can go out of employment as unemployed, housewife, or other. Individuals are censored if they move to education or switch directly to another employment. Finally, individuals who are out of employment can return to employment. Individuals are censored if they move to education. To study the risk of a change in the employment status of individuals by parity among men and women, we estimate three sets of processes: (i) the transition into first employment after leaving full-time education, (ii) the transitions out of employment, and (iii) the transitions into second- and higher-order employment.^2^ Each of these transitions was specified as a hazard function as follows:

1 $$\ln \mu_{i}^{{{\text{FEN}}}} \left( t \right) = \ln \mu_{0} \left( t \right) + \alpha_{1} x_{ij} + \mathop \sum \limits_{j} \alpha_{j} v_{ij} + \mathop \sum \limits_{l} \beta_{l} w_{il} \left( t \right) + \varepsilon_{i}^{{{\text{EN}}}}$$

2 $$\ln \mu_{im}^{{\text{EX}}} \left( t \right) = \ln \mu_{0} \left( t \right) + \alpha_{1} x_{ij} + \mathop \sum \limits_{j} \alpha_{j} v_{ijm} + \mathop \sum \limits_{l} \beta_{l} w_{ilm} \left( t \right) + \varepsilon_{i}^{{\text{EX}}}$$

3 $$\ln \mu_{im}^{{{\text{LEN}}}} \left( t \right) = \ln \mu_{0} \left( t \right) + \alpha_{1} x_{ij} + \mathop \sum \limits_{j} \alpha_{j} v_{ijm} + \mathop \sum \limits_{l} \beta_{l} w_{ilm} \left( t \right) + \varepsilon_{i}^{{{\text{EN}}}}$$

where ${\mu }_{i}^{\text{FEN}}\left(t\right)$ denotes the hazard of first employment entry, ${\mu }_{im}^{\text{EX}}\left(t\right)$ is the hazard of *m*th employment exit and ${\mu }_{im}^{\text{LEN}}\left(t\right)$ is the hazard of *m*th employment entry for individual $i$. Considering Eq. (1), ${\text{ln} \mu }_{0}\left(t\right)$ denotes the baseline log-hazard, which is specified as piecewise constant. The baseline is time (in months) since leaving full-time education. For Eqs. (2) and (3), we estimate multilevel models because each individual can experience several employment changes. For the outcomes of individuals who are out of employment, the baseline is time (in months) since leaving employment; while for the outcomes of employed individuals, the baseline is time (in months) since starting employment. For the three sets of outcomes, our main covariate of interest is parity denoted as ${x}_{ij}$. In addition, ${v}_{ij}$ and ${w}_{il}(t)$ represent time-constant and time-varying characteristics, respectively, that influence individuals’ propensities to change their employment status. Lastly, we include a joint random effect for all employment entries (Eqs. 1 and 3) denoted by ${\varepsilon }_{i}^{\text{EN}}$ and a separate random effect for employment exits (Eq. 2) denoted by ${\varepsilon }_{i}^{\text{EX}}$.

### Joint Model of Employment Changes and Childbearing

Our explanatory variable—childbearing—is likely to be jointly determined with the outcome of interest—employment. To address such concerns, we apply simultaneous-equations hazard models (Aassve et al., 2006; Kulu & Steele, 2013; Lillard & Panis, 1996; Lillard et al., 1995; Matysiak, 2009; Mikolai & Kulu, 2018; Steele et al., 2005, 2006). More specifically, we estimate a joint model of employment changes and childbearing to detect and control for individual-level unobserved factors, which may simultaneously influence both processes. The model is as follows:

4 $$\ln \mu_{i}^{{{\text{FEN}}}} \left( t \right) = \ln \mu_{0} \left( t \right) + \alpha_{1} x_{ij} + \mathop \sum \limits_{j} \alpha_{j} v_{ij} + \mathop \sum \limits_{l} \beta_{l} w_{il} \left( t \right) + \varepsilon_{i}^{{{\text{EN}}}}$$

5 $$\ln \mu_{im}^{{{\text{EX}}}} \left( t \right) = \ln \mu_{0} \left( t \right) + \alpha_{1} x_{ij} + \mathop \sum \limits_{j} \alpha_{j} v_{ijm} + \mathop \sum \limits_{l} \beta_{l} w_{ilm} \left( t \right) + \varepsilon_{i}^{{{\text{EX}}}}$$

6 $$\ln \mu_{im}^{{{\text{LEN}}}} \left( t \right) = \ln \mu_{0} \left( t \right) + \alpha_{1} x_{ij} + \mathop \sum \limits_{j} \alpha_{j} v_{ijm} + \mathop \sum \limits_{l} \beta_{l} w_{ilm} \left( t \right) + \varepsilon_{i}^{{{\text{EN}}}}$$

7 $$\ln \mu_{i}^{C1} \left( t \right) = \ln \mu_{0} \left( t \right) + \alpha_{1} x_{ij} + \mathop \sum \limits_{j} \alpha_{j} v_{ij} + \mathop \sum \limits_{l} \beta_{l} w_{il} \left( t \right) + u_{i}^{C}$$

8 $$\ln \mu_{i}^{C2} \left( t \right) = \ln \mu_{0} \left( t \right) + \alpha_{1} x_{ij} + \mathop \sum \limits_{j} \alpha_{j} v_{ij} + \mathop \sum \limits_{l} \beta_{l} w_{il} \left( t \right)\, + \,u_{i}^{C}$$

9 $$\ln \mu_{i}^{C3} \left( t \right) = \ln \mu_{0} \left( t \right) + \alpha_{1} x_{ij} + \mathop \sum \limits_{j} \alpha_{j} v_{ij} + \mathop \sum \limits_{l} \beta_{l} w_{il} \left( t \right) + u_{i}^{C}$$

where we have three additional equations (Eqs. 7–9). ${\mu }_{i}^{C1}\left(t\right)$ denotes the hazard of a first birth, ${\mu }_{i}^{C2}\left(t\right)$ the hazard of a second birth, and ${\mu }_{i}^{C3}\left(t\right)$ the hazard of a third birth for individual $i$.^3^ Each of the hazard equations included a baseline log-hazard. The baseline log-hazard of first birth was specified as age and the baseline log-hazard of second and third births was specified as time since the birth of the previous child. The hazard equations also include a set of time-constant and time-varying variables among which we have our main covariate of interest—employment. Lastly, each of the hazard equations include a random heterogeneity component which is person-specific. These individual-level random effects aim to control for unmeasured time-constant characteristics that may influence individuals’ likelihood of having a conception or of changing their employment status. We assume that the residuals of the equations follow a joint normal distribution:

10 $$\left( {\begin{array}{*{20}c} {\begin{array}{*{20}c} {\varepsilon_{i}^{{\text{EN}}} } \\ {\varepsilon_{i}^{{\text{EX}}} } \\ \end{array} } \\ {u_{i}^{C} } \\ \end{array} } \right) \sim N\left( {\left( {\begin{array}{*{20}c} 0 \\ 0 \\ 0 \\ \end{array} } \right),\left( {\begin{array}{*{20}c} {\begin{array}{*{20}c} {\sigma_{{\varepsilon_{i}^{{\text{EN}}} }}^{2} } & {\rho_{{\varepsilon_{i}^{{\text{EN}}} \varepsilon_{i}^{{\text{EX}}} }} } & {\rho_{{\varepsilon_{i}^{{\text{EN}}} u_{i}^{C} }} } \\ \end{array} } \\ {\begin{array}{*{20}c} {\begin{array}{*{20}c} {\rho_{{\varepsilon_{i}^{{\text{EX}}} \varepsilon_{i}^{{\text{EN}}} }} } & {\sigma_{{\varepsilon_{i}^{{\text{EX}}} }}^{2} } & {\rho_{{\varepsilon_{i}^{{\text{EX}}} u_{i}^{C} }} } \\ \end{array} } \\ {\begin{array}{*{20}c} {\rho_{{u_{i}^{C} \varepsilon_{i}^{{\text{EN}}} }} } & {\rho_{{u_{i}^{C} \varepsilon_{i}^{{\text{EX}}} }} } & {\sigma_{{u_{i}^{C} }}^{2} } \\ \end{array} } \\ \end{array} } \\ \end{array} } \right)} \right)$$

where ${\sigma }_{{\varepsilon }_{i}^{\text{EN}}}^{2}$, ${\sigma }_{{\varepsilon }_{i}^{\text{EX}}}^{2}$ and ${\sigma }_{{u}_{i}^{C}}^{2}$ denote the variances of the person-specific residuals of the different processes, and $\rho$ is the correlation between the residuals. Identification is ensured by the fact that we observe repeated events, whereas the unobserved heterogeneity components are assumed fixed over individuals’ lifetimes. Although the sources of these unobserved differences may change over time (e.g. due to shifts in preferences or changes in partner or children's characteristics), our model operates under the assumption that such variations do not occur. The potentially endogenous variables always enter the other processes as lagged explanatory variables, which ensures identification of their parameters. Additional exclusion restrictions are not formally required with these restrictions in place (Aassve et al., 2006; Steele et al., 2005).

We estimate two models stepwise. First, we focus on the relationship between parity and employment changes (Model 1). The first model is estimated twice; first (Model 1a) we estimate single-process hazard models for the risk of first employment entry, employment exits, and higher-order employment entries and then (Model 1b) we estimate the multiprocess hazard model where we account for unobserved time-constant co-determinants of the risk of employment entries, employment exits, and the risk of childbirth. Second, we additionally examine whether and the extent to which the effect differs across origin groups (Model 2). In other words, Model 2 includes an interaction term between parity and origin group. Lastly, we report the results of Model 2 when accounting for the unobserved time-constant co-determinants of childbearing and employment risks. For all models, we analyse men and women separately.^4^ Moreover, all models are estimated via maximum likelihood using the aML software (Lillard & Panis, 2003).

An important point to note is that the normal distribution's residuals must be integrated out in hazard models. In multilevel-hazards models, the quality of the estimates can be influenced by the integration points. The results we report in the paper are based on specifying six integration points. However, to ensure the robustness of our results to a different number of integration points, we conducted robustness checks specifying ten integration points and the results remained similar.^5^

### Variables

We include a number of controls in the models. In particular, in the first employment entry equation, we control for time since in education, birth cohort, partnership status, parity, educational level, and origin group. In the employment exits equation, we control for time since previous employment, birth cohort, partnership status, parity, educational level, origin group, order of change, and type of employment. In the higher-order employment entries equation, we control for time since being out of employment, birth cohort, partnership status, parity, educational level, origin group, order of change, and type of out of employment. With regard to childbearing, in the first conception equation, we control for age, birth cohort, partnership status, educational level, origin group, and employment status. Lastly, in the second and third conception equations, we control for time since previous birth, birth cohort, partnership status, educational level, origin group, and employment status. We also conducted additional analyses where we included age at first birth in the second and third conception equations. The results (not reported but available upon request) are similar.

Respondents’ parity status is treated as a time-varying variable using retrospective information on the year and month of each birth, and is categorised as “childless”, “1 child”, “2 children”, and “3 or more children”. For the first employment transition after leaving full-time education, we modify this variable to have only two categories: “childless individuals” and “individuals with children”.^6^ Partnership status is time-varying and is categorised as “single”, “cohabiting”, “married”, and “separated”. The categories “cohabiting” and “married” include both first- and higher-order unions. Birth cohorts include four cohorts: 1948–1959 (reference), 1960–1969, 1970–1979, and 1980–1999. Respondents’ educational level is a time-varying variable and is categorised as low (reference), medium, and high based on the highest diploma obtained by the respondent. The origin group for immigrants’ descendants includes North Africa, sub-Saharan Africa, South East Asia, Turkey, Southern Europe, and other Europe.^7^ Lastly, we control for order of employment change and origin state. Individuals can move into and out of employment several times. Besides, they can move out of employment when being salaried or self-employed and back into employment when being unemployed or housewife for instance.

## Results

Women and men experienced a large number of employment changes and births events. We report the numbers and proportions in Tables 3 and 4 in Appendix. We show the results of two event history models (Models 1–2) of the risk of a change in employment status. To facilitate interpretation, Tables 1 and 2 show the relative risks for the key variables of interest, whereas Tables 5, 6, and 7 in Appendix report log-relative hazards for all variables in the models separately by gender.

**Table 1:** Impact of childbearing on employment, relative risks (Model 1). *Source*: Trajectories and origins, authors’ own calculations

	Women				Men			
	Model 1a single-process		Model 1b multiprocess		Model 1a single-process		Model 1b multiprocess	
	RR	Sig	RR	Sig	RR	Sig	RR	Sig
First employment entry								
Parity								
Childless (ref.)	1		1		1		1	
With children	0.527	***	0.536	***	0.967		0.951	
Origin Group								
Native (ref.)	1		1		1		1	
North Africa	0.710	***	0.713	***	0.909	*	0.922	
Sub-Saharan Africa	0.722	***	0.720	***	0.787	***	0.793	***
South East Asia	0.897		0.902		1.049		1.071	
Turkey	0.655	***	0.654	***	1.560	***	1.655	***
Southern Europe	1.074		1.078		1.327	***	1.359	***
Other Europe	0.815	***	0.810	***	1.063		1.066	
Employment exits								
Parity								
Childless (ref.)	1		1		1		1	
1 child	1.941	***	1.692	***	0.704	***	0.691	***
2 children	2.028	***	1.557	***	0.780	**	0.739	**
3 + children	2.368	***	1.441	***	0.955		1.011	
Origin group								
Native (ref.)	1		1		1		1	
North Africa	1.235	***	1.305	***	1.219	**	1.166	*
Sub-Saharan Africa	0.938		1.075		1.143		1.132	
South East Asia	0.973		0.986		1.055		1.000	
Turkey	1.570	***	1.761	***	1.030		0.919	
Southern Europe	0.851	**	0.810	***	0.824	**	0.747	***
Other Europe	1.108		1.158		1.177		1.082	
Higher-order employment entries								
Parity								
Childless (ref.)	1		1		1		1	
1 child	0.595	***	0.607	***	0.792	***	0.799	***
2 children	0.488	***	0.495	***	0.641	***	0.652	***
3 + children	0.379	***	0.377	***	0.681	***	0.646	***
Origin group								
Native (ref.)	1		1		1		1	
North Africa	0.894		0.890		0.772	**	0.750	**
Sub-Saharan Africa	0.863		0.839		0.721	**	0.682	**
South East Asia	1.002		1.023		0.689	**	0.695	**
Turkey	0.783		0.739	*	0.957		1.031	
Southern Europe	1.050		1.084		0.864		0.890	
Other Europe	0.957		0.945		0.846		0.838	
Unobserved Heterogeneity								
Standard deviation of residuals								
Fertility	0.726	***	0.730	***	0.729	***	0.732	***
Employment entry	0.618	***	0.697	***	0.656	***	0.795	***
Employment exit	1.130	***	1.307	***	0.885	***	1.205	***
Correlation between residuals								
Fertility and employment entry			− 0.114	***			0.095	*
Fertility and employment exit			0.302	***			− 0.070	
Employment entry and exit			− 0.658	***			− 0.962	***

See Table 4 in Appendix for the results of the full equations.

*p < 0.1; **p < 0.05; ***p < 0.01

**Table 2:** Impact of childbearing on employment by origin group, relative risks (Model 2). *Source*: Trajectories and origins, authors’ own calculations

	Women		Men	
	Model 2 multiprocess		Model 2 multiprocess	
	RR	Sig	RR	Sig
Effects of parity status on employment exits by origin group				
Childless × North Africa	1.297	**	0.913	
Childless × Sub-Saharan Africa	1.083		0.725	*
Childless × South East Asia	1.125		0.946	
Childless × Turkey	1.344		0.825	
Childless × Southern Europe	0.897		0.775	***
Childless × Other Europe	1.022		1.099	
Childless × Natives (ref.)	1		1	
1 + children × North Africa	1.859	***	0.927	
1 + children × Sub-Saharan Africa	1.408	**	1.010	
1 + children × South East Asia	1.383	*	0.578	*
1 + children × Turkey	2.875	***	1.027	
1 + children × Southern Europe	1.331	***	0.498	***
1 + children × Other Europe	1.853	***	0.559	***
1 + children × Natives	1.723	***	0.448	***
Effects of parity status on later employment entries by origin group				
Childless × North Africa	1.218		0.845	
Childless × Sub-Saharan Africa	0.884		0.760	
Childless × South East Asia	1.369	*	0.713	**
Childless × Turkey	1.213		0.837	
Childless × Southern Europe	1.395	***	0.852	
Childless × Other Europe	1.274		0.952	
Childless × Natives (ref.)	1		1	
1 + children × North Africa	0.710	***	0.558	***
1 + children × Sub-Saharan Africa	0.978		0.687	
1 + children × South East Asia	0.820		0.596	*
1 + children × Turkey	0.527	***	0.882	
1 + children × Southern Europe	0.814		0.652	***
1 + children × Other Europe	0.778		0.530	***
1 + children × Natives	0.628		0.658	
Unobserved heterogeneity				
Standard deviation of residuals				
Fertility	0.731	***	0.709	***
Employment entry	0.681	***	0.796	***
Employment exit	1.325	***	1.249	***
Correlation between residuals				
Fertility and employment entry	− 0.290	***	0.044	
Fertility and employment exit	0.312	***	0.016	
Employment entry and exit	− 0.665	***	− 0.937	***

See Table 6 in Appendix for the results of the full equations

^*^p < 0.1; **p < 0.05; ***p < 0.01

### Selection

We first estimate single-process hazard models for the risk of first employment entry, employment exits, and higher-order employment entries. We then model jointly the risk of a change in the employment status and the risk of having a birth. The results are reported in Table 1. We start our discussion by focusing on the estimates of the standard deviations of the unobserved heterogeneity terms and their pairwise correlations. The standard deviations of the person-specific residuals are significant in all models. This implies that there is a significant portion of individual-specific heterogeneity that is not accounted for by our covariates. It represents an individual-specific propensity to have children in the fertility equations and an individual-specific propensity to work in the employment equations. These results provide a justification for the need to estimate the multiprocess hazard model.

The person-specific unobserved heterogeneity terms are correlated. This means that the hazards of birth and employment changes have unobserved co-determinants. Our results show that, for women, the unobserved characteristics that increase the propensity to have a child are negatively correlated with the unobserved characteristics that increase the propensity to enter employment and positively correlated with the unobserved characteristics that increase the propensity to exit employment. We do not find significant correlations for men. These findings indicate that women who are more likely to have a child are more likely to leave employment and less likely to re-enter the labour market. Due to the unobserved selection, the estimates of the impacts of fertility on employment obtained from the single-process hazard models are slightly biased, especially on the impact of children on employment exits.

### Impact of Childbearing on Employment

Our findings (reported in Table 1, Models 1a and 1b) show first some heterogeneity in employment patterns across origin groups. Among women, all descendant groups except the female descendants of Southern European immigrants are less likely to enter their first employment spell compared to natives. Some descendant groups such as the descendants of North African and Turkish immigrants are also more likely to go out of employment. Among men, the male descendants of sub-Saharan African immigrants are less likely than other groups to enter their first employment spell. We also find that the male descendants of sub-Saharan African, North African, and South East Asian immigrants are less likely to experience higher-order employment entries. The male descendants of North African immigrants are more likely to exit employment, as opposed to the male descendants of Southern European immigrants who are less likely to go out of the labour market.

Regarding parity, children have a strong and clearly negative impact on women’s employment. Mothers are less likely to take up a job than childless women do. They are also more likely to exit employment following childbirth. We also find that as parity increases, women are less likely to re-enter employment. Yet, the likelihood of exiting employment decreases as parity increases. Regarding men, we find mixed results: while men are less likely to enter employment following a birth, they are also less likely to exit employment compared to childless men.

These conclusions can be drawn from the estimates obtained from the single-process models as well as those from the multiprocess hazard model. However, comparing the results of the two models reveals that controlling for unobserved co-determinants reduces the negative impact of parity on women’s employment. In other words, the negative effect of childbirth on the risk of exiting or re-entering employment is overestimated (although slightly) for women if we do not account for unobserved co-determinants of these two processes. For men, we find that the negative effect of parity on employment entries is marginally overestimated, although caution is needed when interpreting the results for men given that they change between models.

Next, we examine possible differences across origin groups in the effect of parity on the risks of employment entry and exit for both men and women (Table 2). To ensure a reasonable number of observations, we regroup all individuals who have children under one category. Furthermore, it is important to note that, due to limited sample size, the transition to first employment does not include an interaction term between parity and origin group. The results show that, among women, all groups are negatively affected by childbirth; yet the female descendants of Turkish immigrants are the least likely to enter employment after childbirth and the most likely to exit employment following childbirth. Among men, there is more heterogeneity: while the French natives as well as the descendants of European immigrants are less likely to exit employment after having a child, we do not find any significant relationship between childbirth and employment exits for other descendant groups.

## Conclusion and Discussion

This paper investigated the association between family formation and the labour market changes of immigrants’ descendants in France. We examined whether and how the association between fertility and employment differs by gender and migration background. We contribute to the literature by simultaneously investigating the effect of childbearing on all employment entries and exits in family ages among both female and male descendants of immigrants. Another novelty is that we also addressed the issue of selection on unobserved characteristics, i.e. individuals who are more likely to have a child (or children) are more/less likely to enter or exit employment. We applied a multiprocess hazard model that allows a correlation of person-specific error terms of fertility and employment transitions.

In line with our first hypothesis, our analysis reveals a strong negative impact of childbearing on women’s work. The arrival of the first child reduces the propensity of employment entry and increases the risk of employment exit. Furthermore, higher-order births further reduce women’s likelihood of re-entering the labour market. We do not find evidence of a stronger negative impact of childbirth on the employment of female descendants of immigrants compared to native women (Hypothesis 2). However, the effect of family formation differs among immigrants’ descendants, which supports our third hypothesis. Although all groups are negatively affected, the female descendants of Turkish immigrants are the most likely to exit employment and the least likely to re-enter employment following childbirth. Lastly, we have found that not controlling for unobservables led to a small overestimation of the negative effect of childbearing on women’s work, confirming our fourth hypothesis. Yet, the negative effect remains significant even after controlling for these unobservables.

We expected to find no significant association between childbirth and men’s work. Yet, we find that having a child also affects the employment trajectories of men: fathers are less likely to exit employment but also less likely to re-enter employment compared to childless men. The direction of the effect differs across origin groups: while the French natives as well as the male descendants of European immigrants are less likely to exit employment after having a child, we do not find any significant association between childbirth and employment exits for other descendant groups. Regarding the lower likelihood of employment re-entry for fathers compared to childless men, previous studies have shown, for instance, that past unemployment increases the probability of current unemployment. This has been found to be the case particularly among second-generation Middle-Eastern, Turkish, and Southern European immigrants in Sweden (Aradhya et al. 2023). It might be that given the difficulty of re-entering the labour market due to a history of prolonged unemployment spells, fathers are less likely to rejoin the labour market. Our results thus suggest that the association between childbirth and men’s work is more complicated.

There are a number of potential explanations for the negative association between childbirth and employment among women. First, there might be differences in the use of childcare among individuals with a migration background compared to the native population. For instance, the descendants of immigrants may hold an aversion towards formal childcare due to institutional distrust. This was reported by second-generation Moroccan immigrant mothers in Flanders due to negative experiences such as discrimination as a school pupil, or negative experiences as a childcare worker (Wood, 2022). The descendants of immigrants may also hold different preferences regarding child upbringing compared to natives (Eremenko & Unterreiner, 2023; Guirola & Sánchez-Domínguez, 2022; Leitner, 2003; Saraceno & Keck, 2010). These variations in the use of care could explain why immigrants’ descendants from certain groups have distinct labour market trajectories upon childbirth compared to the natives. More research is needed to understand whether this is at play.

Another reason that could explain our result is that there is also a high instability of employment contracts especially among minority populations (Algan et al., 2010; Meurs et al., 2006). A history of unstable employment prior to family formation is likely to influence individuals’ decision to remain or not in the labour market upon childbirth (Maes et al., 2021). A history of unemployment might also decrease their ability to rejoin the labour market (Aradhya et al. 2023). This has implications for access to family policies. Among other things, it affects, for instance, the allowance that parents who partially/totally stop working in the labour market to look after their children are entitled to. Therefore, having an unstable position or poor employment conditions/prospects might explain why immigrants’ descendants are more prone to leave the labour market upon childbirth compared to natives.

On a similar note, the descendants of immigrants are more prone to experience discrimination in the labour market compared to the native population (Bertrand & Duflo, 2017; Di Stasio et al., 2021; Lippens et al., 2023; Quillian & Midtbøen, 2021; Quillian et al., 2019). In France, a large body of research has found that the descendants of immigrants from sub-Saharan Africa, North Africa, and Turkey are more likely to experience labour market discrimination (Meurs et al., 2006). There are also substantial gender disparities in employment and discrimination (Di Stasio & Larsen, 2020; Petit et al., 2013). As a result of this, the children of immigrants may become demotivated to continue to work and may consider family formation as a suitable alternative career.

Lastly, social norms are likely to play an important role. Although the descendants of immigrants have been socialised in an egalitarian family context in France, they might also remain influenced by parental attitudes, family networks, and the wider migrant community (Diehl et al., 2009; Glas, 2023; Idema & Phalet, 2007; Inglehart & Norris, 2003; Khoudja & Fleischmann, 2017; Moriconi & Rodríguez-Planas, 2021; Sanchez Guerrero & Schober, 2021). Previous studies have shown that some groups among the second generation of immigrants in France continue to exhibit similar family patterns than those of their parents rather than those of natives (Delaporte & Kulu, 2022; Kulu et al., 2021; Pailhé, 2015, 2017). Therefore, individuals who come from countries with more traditional values might hold more strongly the perception that women are the main homemakers and care providers. This could explain why we find that female descendants of Turkish immigrants are less likely to enter employment and more likely to exit employment after a birth.

Overall, our study contributes to the existing body of literature on gender and immigration by analysing the interaction effect of parenthood, and migration background on women’ and men’ labour market trajectories in France. By adopting an intersectional perspective, we are able to analyse how family and employment interact among both men and women from different migration background in terms of nativity but also migrant origin. We also adopt a life-course perspective and study repeated events of employment changes. Lastly, we adopt a simultaneous-equations modelling approach which allows us to detect and control for unobserved time-constant co-determinants of these two processes. Our study sheds light on how differently immigrant descendant and native men and women reconcile work with family life in France and highlights the need for policies to encourage women to participate in the labour market and to remain economically active following childbirth.

Yet, while our study offers valuable insights, it is important to acknowledge its limitations. One of them concerns the data employed. Utilising administrative data would significantly enhance our research, as it enables the disaggregation of analysis by specific countries of origin. This approach would allow for a more detailed examination of a broader array of origin groups. Additionally, administrative datasets typically encompass larger sample sizes, which mitigates the challenges often encountered with smaller, less representative samples. Some questions also remain unanswered. For instance, incorporating information regarding the origin background and the employment history of the partner into the analysis to examine how this influences the other spouse’ labour supply upon childbirth could be a fruitful avenue for future research.

## Figures and Tables

**Fig. 1:**
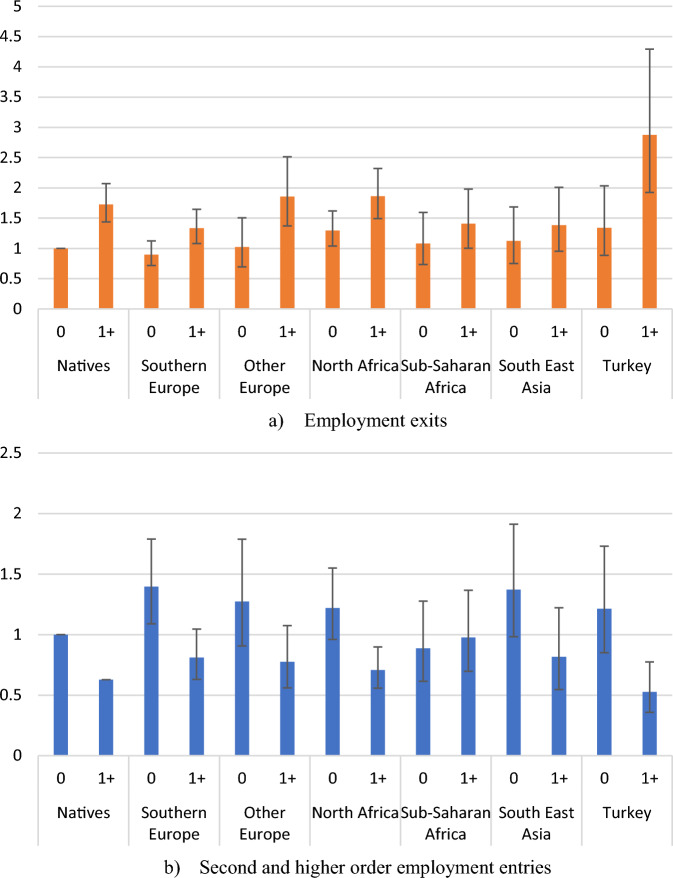
Relative risks of employment entry and exit by origin and parity for women (Model 2). Source: Trajectories and Origins, authors’ own calculations

**Fig. 2:**
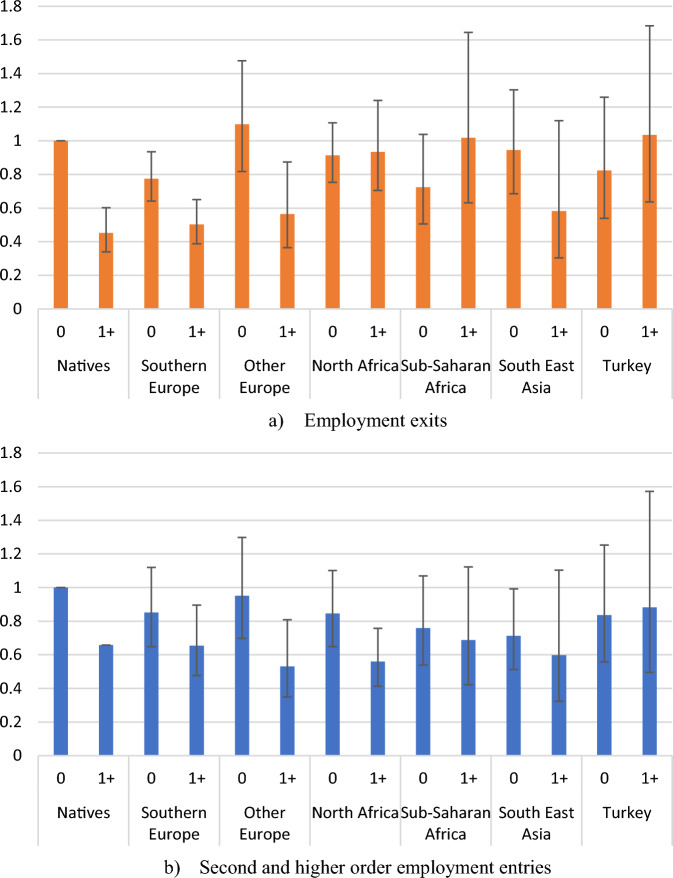
Relative risks of employment entry and exit by origin and parity for men (Model 2). Source: Trajectories and Origins, authors’ own calculations

## Data Availability

The codes to reproduce the analysis are available upon request but the data is not as access must be requested directly to the producer.

## Appendix

See Figs. 1 and 2.

See Tables

**Table 3:** Number and proportion of person-months and events by sets of outcomes and categories of variables. *Source:* Trajectories and origins, authors’ own calculations

	Women				Men			
	Person-months		Events		Person-Months		Events	
	Number	%	Number	%	Number	%	Number	%
First employment entry								
Time since in education								
0–1 year	29,411	17	4165	72	27,609	22	3740	69
1–3 years	33,260	19	789	14	25,325	20	1014	19
3–5 years	22,223	13	278	5	14,379	12	240	4
5+ years	92,209	52	515	9	56,247	46	401	7
Birth cohort								
1948–1959	36,247	20	823	14	13,407	11	757	14
1960–1969	66,290	37	1689	29	49,984	40	1486	28
1970–1979	49,472	28	2005	35	41,749	34	1811	34
1980–1999	25,094	14	1230	21	18,420	15	1341	25
Partnership status								
Single	64,951	37	3650	64	76,688	62	4183	78
Cohabiting	23,125	13	947	16	15,609	13	638	12
Married	73,354	41	861	15	23,090	19	413	8
Separated	15,674	9	289	5	8174	7	161	3
Parity								
Childless	83,826	47	4993	87	94,846	77	5008	93
With children	93,277	53	754	13	28,715	23	387	7
Educational level								
Low	80,075	45	861	15	36,561	30	946	18
Medium	78,058	44	2964	52	62,085	50	2979	55
High	18,970	11	1922	33	24,913	20	1470	27
Origin group								
Native	35,554	20	1592	28	33,534	27	1386	26
North Africa	58,930	33	1229	21	32,612	26	1064	20
Sub-Saharan Africa	13,693	8	360	6	8449	7	330	6
South East Asia	6994	4	320	6	6887	6	342	6
Turkey	10,946	6	220	4	3906	3	272	5
Southern Europe	31,313	18	1440	25	26,006	21	1446	27
Other Europe	13,889	8	388	7	7910	6	388	7
Total	177,103	100	5747	100	123,560	100	5395	100
Employment exits								
Time since previous employment								
0–1 year	90,773	11	329	12	86,965	10	223	12
1–3 years	145,729	17	693	26	138,185	16	618	34
3–5 years	113,854	14	461	17	107,987	13	326	18
5+ years	484,327	58	1211	45	509,486	60	626	35
Birth cohort								
1948–1959	235,795	28	559	21	259,957	31	401	22
1960–1969	338,041	40	938	35	321,567	38	605	34
1970–1979	217,752	26	914	34	206,696	25	552	31
1980–1999	43,095	5	283	11	54,404	6	235	13
Partnership status								
Single	196,925	24	459	17	246,979	29	900	50
Cohabiting	135,400	16	473	18	139,148	17	250	14
Married	387,062	46	1413	52	372,557	44	404	23
Separated	115,296	14	349	13	83,939	10	239	13
Parity								
Childless	348,665	42	861	32	405,428	48	1230	69
1 child	197,950	24	802	30	158,201	19	199	11
2 children	202,354	24	677	25	180,194	21	207	12
3+ children	85,714	10	354	13	98,800	12	157	9
Educational level								
Low	144,390	17	640	24	186,593	22	524	29
Medium	449,340	54	1616	60	477,981	57	1038	58
High	240,953	29	438	16	178,049	21	231	13
Origin group								
Native	281,032	34	783	29	260,609	31	460	26
North Africa	141,905	17	605	22	136,815	16	413	23
Sub-Saharan Africa	31,325	4	111	4	31,645	4	88	5
South East Asia	34,527	4	105	4	36,604	4	88	5
Turkey	17,759	2	136	5	27,500	3	71	4
Southern Europe	244,096	29	683	25	268,906	32	478	27
Other Europe	61,979	7	181	7	62,600	7	138	8
Order								
1	672,962	81	1934	72	632,158	75	1289	72
2	114,258	14	558	21	139,062	17	335	19
3+	47,463	6	202	7	71,403	8	169	9
Type of employment								
Salaried	799,304	96	2609	97	772,092	92	1708	95
Self-employed	35,379	4	85	3	70,531	8	85	5
Total	834,683	100	2694	100	842,623	100	1793	100
Higher-order employment entries								
Time since out of employment								
0–1 year	59,192	16	672	22	47,244	20	822	29
1–3 years	76,334	21	1017	33	45,062	19	1018	36
3–5 years	48,697	13	383	12	27,235	12	218	8
5+ years	180,544	49	993	32	117,010	49	736	26
Birth cohort								
1948–1959	109,673	30	532	17	47,283	20	385	14
1960–1969	140,282	38	1076	35	101,317	43	989	35
1970–1979	86,609	24	1044	34	68,418	29	1052	38
1980–1999	28,203	8	413	13	19,533	8	368	13
Partnership status								
Single	70,985	19	835	27	98,007	41	1367	49
Cohabiting	42,852	12	498	16	36,146	15	522	19
Married	207,177	57	1280	42	77,944	33	654	23
Separated	43,753	12	452	15	24,454	10	251	9
Parity								
Childless	97,934	27	1309	43	139,391	59	1970	71
1 child	74,029	20	645	21	33,990	14	305	11
2 children	96,780	27	668	22	40,770	17	329	12
3+ children	96,024	26	443	14	22,401	9	190	7
Educational level								
Low	133,274	37	659	22	70,825	30	586	21
Medium	193,143	53	1801	59	131,732	56	1590	57
High	38,350	11	605	20	33,994	14	618	22
Origin group								
Native	104,789	29	793	26	69,033	29	757	27
North Africa	86,682	24	705	23	51,746	22	588	21
Sub-Saharan Africa	16,387	4	164	5	10,507	4	146	5
South East Asia	12,877	4	145	5	12,116	5	150	5
Turkey	16,432	5	132	4	6334	3	95	3
Southern Europe	89,752	25	798	26	62,479	26	726	26
Other Europe	27,827	8	220	7	18,795	8	235	8
Order								
1	273,396	75	2195	72	175,814	74	2050	73
2	68,643	19	626	20	48,379	20	550	20
3+	22,728	6	244	8	12,358	5	194	7
Type of out of employment								
Unemployed	73,818	20	1209	39	60,107	25	1043	37
Housewife	202,147	55	1102	36	2076	1	20	0.7
Other	88,802	24	754	25	174,368	74	1731	62
Total	364,767	100	3065	100	236,551	100	2794	100

The proportions by origin group do not equal to 100 when we sum up because there is around 3% of the sample that belong to other smaller origin groups

3,

**Table 4:** Number and proportion of person-months and events by sets of outcomes and categories of variables. *Source:* Trajectories and Origins, authors’ own calculations

	Women				Men			
	Person-months		Events		Person-months		Events	
	Number	%	Number	%	Number	%	Number	%
First conception								
Age								
15–19	432,585	44	599	14	401,852	39	129	4
20–24	293,506	30	1,750	40	305,411	30	923	28
25–29	137,728	14	1,440	33	172,850	17	1,384	43
30–34	54,954	6	468	11	74,510	7	625	19
35+	53,750	6	115	3	63,737	6	190	6
Birth cohort								
1948–1959	132,116	14	753	17	141,519	14	669	21
1960–1969	273,985	28	1,545	35	291,052	29	1,226	38
1970–1979	319,226	33	1,587	36	329,635	32	1,147	35
1980–1999	247,197	25	487	11	256,122	25	209	6
Partnership status								
Single	742,545	76	721	16	805,263	79	446	14
Cohabiting	101,992	10	1,200	27	98,952	10	964	30
Married	80,836	8	2,285	52	62,228	6	1,705	52
Separated	47,151	5	166	4	51,885	5	136	4
Educational level								
Low	440,943	45	1,028	24	462,941	45	703	22
Medium	392,441	40	2,283	52	436,094	43	1,842	57
High	139,140	14	1,061	24	119,292	12	706	22
Origin group								
Native	252,575	30	1,254	29	259,603	25	921	28
North Africa	219,542	23	918	21	210,187	21	591	18
Sub-Saharan Africa	71,571	7	241	6	69,024	7	159	5
South East Asia	62,093	6	207	5	77,759	8	150	5
Turkey	35,070	4	180	4	36,557	4	152	5
Southern Europe	226,771	23	1,119	26	255,519	25	956	29
Other Europe	67,563	7	320	7	73,172	7	240	7
Employment status								
Abroad	8,187	0.8	38	0.9	8,047	0.8	23	0.7
Salaried	318,148	33	2,812	64	367,528	36	2,234	69
Self-employed	8,835	0.9	62	1	17,585	2	132	4
Unemployed	24,581	3	153	3	25,415	2	57	2
Student	537,326	55	599	14	483,873	48	238	7
Housewife	14,572	1	323	7	1,059	0.1	2	0
Inactive	39,155	4	249	6	67,547	7	193	6
Other	21,718	2	136	3	47,274	5	372	11
Total	972,524	100	4,372	100	1,018,328	100	3,251	100
Second conception								
Time since previous birth								
0–1 year	50,314	19	312	10	37,305	20	220	10
1–3 years	72,883	27	1,526	50	52,913	29	1,168	52
3–5 years	38,824	15	722	24	26,404	14	509	23
5+ years	103,595	39	506	17	66,516	36	354	16
Birth cohort								
1948–1959	69,046	26	599	20	58,673	32	537	24
1960–1969	111,378	42	1,185	39	73,457	40	965	43
1970–1979	70,189	26	1,102	36	45,321	25	688	31
1980–1999	15,003	6	180	6	5,687	3	61	3
Age at birth 1								
16–19	24,115	9	368	12	5,932	3	72	3
20–24	108,131	41	1,282	42	49,181	27	649	29
25–29	87,047	33	1,059	35	77,618	42	993	44
30–34	34,067	13	292	10	37,898	21	429	19
35+	10,684	4	42	1	11,856	6	102	5
Partnership status								
Single	22,435	8	132	4	8522	5	56	2
Cohabiting	51,422	19	580	19	43,157	24	483	21
Married	142,247	54	2,168	71	107,648	59	1,611	72
Separated	49,513	19	186	6	23,810	13	101	4
Educational level								
Low	59,441	22	671	22	42,037	23	478	21
Medium	149,062	56	1,645	54	109,083	60	1,254	56
High	57,113	22	750	24	32,018	17	519	23
Origin group								
Native	86,799	33	902	29	59,385	32	642	29
North Africa	47,266	18	641	21	26,163	14	406	18
Sub-Saharan Africa	12,560	5	143	5	6,473	4	104	5
South East Asia	11,356	4	133	4	5,692	3	100	4
Turkey	6,282	2	129	4	5,552	3	115	5
Southern Europe	72,829	27	817	27	60,396	33	676	30
Other Europe	21,404	8	215	7	14,930	8	161	7
Employment status								
Abroad	1,113	0.4	22	0.7	798	0.4	16	0.7
Salaried	171,763	65	1,882	61	128,451	70	1,604	71
Self-employed	5,816	2	59	2	11,000	6	152	7
Unemployed	9,059	3	69	2	3,458	2	31	1
Student	9,759	4	81	3	4,803	3	51	2
Housewife	32,350	12	643	21	172	0	4	0
Inactive	12,999	5	128	4	6,779	4	70	3
Other	22,757	9	182	6	27,677	15	323	14
Total	265,615	100	3,066	100	183,138	100	2,251	100
Third conception								
Time since previous birth								
0–1 year	36,336	12	129	11	26,654	12	94	11
1–3 years	59,297	20	520	43	43,811	20	371	43
3–5 years	42,638	14	308	25	31,088	14	221	25
5+ years	161,913	54	266	22	115,987	53	182	21
Birth cohort								
1948–1959	106,320	35	286	23	89,583	41	236	27
1960–1969	130,934	44	492	40	94,046	43	399	46
1970–1979	57,098	19	411	34	32,422	15	221	25
1980–1999	5,833	2	34	3	1,489	0.7	12	1
Age at birth 1								
16–19	34,154	11	236	19	5,565	3	50	6
20–24	139,669	40	588	48	76,662	35	316	36
25–29	99,872	33	324	26	95,921	44	338	39
30–34	21,763	7	52	4	31,898	15	137	16
35+	3,082	1	7	0.6	6,433	3	23	3
Partnership status								
Single	8,179	3	33	3	2,741	1	14	2
Cohabiting	32,416	11	139	11	28,298	13	129	15
Married	216,455	72	956	78	165,360	76	662	76
Separated	43,135	14	95	8	21,140	10	63	7
Educational level								
Low	64,919	22	376	31	45,474	21	246	28
Medium	172,683	58	613	50	128,040	59	447	51
High	62,583	21	234	19	44,026	20	175	20
Origin group								
Native	112,153	37	330	27	77,172	35	221	25
North Africa	40,357	13	329	27	24,578	11	207	24
Sub-Saharan Africa	7,924	3	66	5	5,746	3	54	6
South East Asia	8,541	3	54	4	7,494	3	42	5
Turkey	6,540	2	67	5	5,576	3	58	7
Southern Europe	96,956	32	242	20	77,841	36	207	24
Other Europe	20,690	7	98	8	15,333	7	65	7
Employment status								
Abroad	935	0.3	13	1	596	0.3	6	0.7
Salaried	180,233	60	542	44	146,481	67	619	71
Self-employed	11,170	4	24	2	20,986	10	59	7
Unemployed	7,080	2	30	2	3,365	2	16	2
Student	3,397	1	16	1	2,288	1	17	2
Housewife	50,059	17	442	36	218	0.1	2	0.2
Inactive	11,439	4	59	5	5,592	3	33	4
Other	35,872	12	97	8	38,014	17	116	13
Total	300,185	100	1223	100	217,539	100	868	100

The proportions by origin group do not equal to 100 when we sum up because there is around 3% of the sample that belong to other smaller origin groups

4,

**Table 5:** Impact of childbearing on employment, log-relative risks (Model 1, full specification)*. * *Source:* Trajectories and origins, authors’ own calculations

	Women				Men			
	Model 1a single-process		Model 1b multi-process		Model 1a Single-process		Model 1b Multi-process	
	RR	Sig	RR	Sig	RR	Sig	RR	Sig
First employment entry								
Constant	− 1.293	***	− 1.364	***	− 1.108	***	− 1.212	***
Time Since in Education								
0–1 year (slope)	− 0.260	***	− 0.252	***	− 0.216	***	− 0.203	***
1–3 years (slope)	0.032	***	0.031	***	0.025	***	0.027	***
3–5 years (slope)	− 0.026	***	− 0.025	***	− 0.061	***	− 0.058	***
5+ years (slope)	0.001		0.001		0.004	***	0.005	***
Birth cohort								
1948–1959 (ref.)	0		0		0		0	
1960–1969	− 0.246	***	− 0.238	***	− 0.455	***	− 0.471	***
1970–1979	− 0.217	***	− 0.200	***	− 0.434	***	− 0.443	***
1980–1999	− 0.357	***	− 0.340	***	− 0.296	***	− 0.274	***
Partnership status								
Single (ref.)	0		0		0		0	
Cohabiting	0.095	**	0.080		0.389	***	0.413	***
Married	− 0.187	***	− 0.223	***	0.281	***	0.308	***
Separated	0.350	***	0.338	***	0.136		0.189	*
Parity								
Childless (ref.)	0		0		0		0	
With children	− 0.641	***	− 0.624	***	− 0.034		− 0.050	
Educational level								
Low (ref.)	0		0		0		0	
Medium	0.746	***	0.773	***	0.358	***	0.373	***
High	1.138	***	1.179	***	0.391	***	0.430	***
Origin group								
Native (ref.)	0		0		0		0	
North Africa	− 0.342	***	− 0.338	***	− 0.095	*	− 0.081	
Sub-Saharan Africa	− 0.326	***	− 0.328	***	− 0.239	***	− 0.232	***
South East Asia	− 0.109		− 0.103		0.048		0.069	
Turkey	− 0.423	***	− 0.425	***	0.− 445	***	0.504	***
Southern Europe	0.071		0.075		0.283	***	0.307	***
Other Europe	− 0.205	***	− 0.211	***	0.061		0.064	
Employment exits								
Constant	− 9.977	***	− 9.919	***	− 8.961	***	− 8.994	***
Time since previous employment								
0–1 year (slope)	0.313	***	0.317	***	0.329	***	0.332	***
1–3 years (slope)	− 0.021	***	− 0.018	***	− 0.044	***	− 0.040	***
3+ years (slope)	− 0.001	**	0.001		0.001	***	0.002	***
Birth cohort								
1948–1959 (ref.)	0		0		0		0	
1960–1969	0.258	***	0.297	***	0.173	*	0.335	***
1970–1979	0.749	***	0.796	***	0.472	***	0.615	***
1980–1999	1.365	***	1.487	***	0.610	***	0.885	***
Partnership status								
Single (ref.)	0		0		0		0	
Cohabiting	0.346	***	0.403	***	− 0.508	***	− 0.536	***
Married	0.482	***	0.643	***	− 0.875	***	− 0.901	***
Separated	0.275	***	0.323	***	− 0.004		− 0.089	
Parity								
Childless (ref.)	0		0		0		0	
1 child	0.663	***	0.526	***	− 0.351	***	− 0.370	***
2 children	0.707	***	0.443	***	− 0.249	**	− 0.303	**
3+ children	0.862	***	0.365	***	− 0.046		0.011	
Educational level								
Low (ref.)	0		0		0		0	
Medium	− 0.298	***	− 0.512	***	− 0.326	***	− 0.450	***
High	− 1.194	***	− 1.495	***	− 0.901	***	− 1.084	***
Origin group								
Native (ref.)	0		0		0		0	
North Africa	0.211	***	0.266	***	0.198	**	0.154	*
Sub-Saharan Africa	− 0.064		0.072		0.134		0.124	
South East Asia	− 0.027		− 0.014		0.054		− 0.001	
Turkey	0.451	***	0.566	***	0.030		− 0.084	
Southern Europe	− 0.161	**	− 0.211	***	− 0.194	**	− 0.292	***
Other Europe	0.103		0.147		0.163		0.079	
Order of change								
1 (ref.)	0		0		0		0	
2	0.095		0.293	***	0.181	***	0.317	***
3+	− 0.614	***	− 0.272	**	− 0.046		0.241	**
Type of employment								
Salaried (ref.)	0		0		0		0	
Self-employed	− 0.412	***	− 0.432	***	− 0.436	***	− 0.447	***
Higher-order employment entries								
Constant	− 7.765	***	− 7.718	***	− 8.876	***	− 8.792	***
Time since out of employment								
0–1 year (slope)	0.312	***	0.315	***	0.488	***	0.498	***
1–3 years (slope)	− 0.020	***	− 0.019	***	− 0.081	***	− 0.076	***
3–5 years (slope)	− 0.012	***	− 0.011	***	− 0.018	***	− 0.018	***
5+ years (slope)	0.005	***	0.005	***	0.008	***	0.009	***
Birth cohort								
1948–1959 (ref.)	0		0		0		0	
1960–1969	0.290	***	0.282	***	0.176	**	0.127	*
1970–1979	0.618	***	0.612	***	0.444	***	0.387	***
1980–1999	0.768	***	0.743	***	0.564	***	0.542	***
Partnership status								
Single (ref.)	0		0		0		0	
Cohabiting	0.032		0.036		0.227	***	0.337	***
Married	− 0.221	***	− 0.224	***	− 0.163	**	− 0.006	
Separated	0.079		0.088		− 0.240	***	− 0.113	
Parity								
Childless (ref.)	0		0		0		0	
1 child	− 0.519	***	− 0.499	***	− 0.233	***	− 0.225	***
2 children	− 0.717	***	− 0.703	***	− 0.445	***	− 0.428	***
3+ children	− 0.969	***	− 0.975	***	− 0.384	***	− 0.437	***
Educational level								
Low (ref.)	0		0		0		0	
Medium	0.567	***	0.636	***	0.517	***	0.575	***
High	1.179	***	1.325	***	1.101	***	1.158	***
Origin group								
Native (ref.)	0		0		0		0	
North Africa	− 0.112		− 0.117		− 0.259	**	− 0.288	**
Sub-Saharan Africa	− 0.147		− 0.176		− 0.327	**	− 0.382	**
South East Asia	0.002		0.023		− 0.373	**	− 0.364	**
Turkey	− 0.245		− 0.303	*	− 0.044		0.031	
Southern Europe	0.049		0.081		− 0.146		− 0.117	
Other Europe	− 0.044		− 0.057		− 0.167		− 0.177	
Order of change								
1 (ref.)	0		0		0		0	
2	0.402	***	0.463	***	0.249	***	0.299	***
3+	0.527	***	0.692	***	0.293	***	0.488	***
Type of out of employment								
Unemployed (ref.)	0		0		0		0	
Housewife	− 0.732	***	− 0.766	***	− 0.523	*	− 0.543	*
Other	− 0.554	***	− 0.626	***	− 0.330	***	− 0.361	***
First conception								
Constant	− 7.039	***	− 7.025	***	− 9.309	***	− 9.318	***
Age								
15–19 (slope)	0.014	***	0.015	***	0.040	***	0.040	***
20–24 (slope)	0.001		0.001		0.001		0.001	
25–29 (slope)	0.005	***	0.005	***	0.007	***	0.008	***
30–34 (slope)	− 0.004	**	− 0.004	**	− 0.003	**	− 0.003	**
35+ (slope)	− 0.024	***	− 0.024	***	− 0.016	***	− 0.016	***
Birth cohort								
1948–1959 (ref.)	0		0		0		0	
1960–1969	0.126	**	0.136	**	− 0.017		− 0.027	
1970–1979	− 0.019		− 0.005		− 0.279	***	− 0.288	***
1980–1999	− 0.287	***	− 0.266	***	− 0.710	***	− 0.723	***
Partnership status								
Single (ref.)	0		0		0		0	
Cohabiting	2.249	***	2.259	***	2.479	***	2.478	***
Married	3.251	***	3.261	***	3.644	***	3.642	***
Separated	1.166	***	1.171		1.173	***	1.174	***
Educational level								
Low (ref.)	0		0		0		0	
Medium	− 0.302	***	− 0.313	***	− 0.091		− 0.084	
High	− 0.592	***	− 0.613	***	− 0.362	***	− 0.350	***
Origin group								
Native (ref.)	0		0		0		0	
North Africa	− 0.028		− 0.018		0.117	*	0.119	*
Sub-Saharan Africa	0.320	***	0.322	***	0.303	***	0.303	***
South East Asia	− 0.073		− 0.069		− 0.040		− 0.037	
Turkey	0.001		0.008		0.472	***	0.486	***
Southern Europe	− 0.101	*	− 0.105	**	0.098	*	0.103	*
Other Europe	0.017		0.022		− 0.107		− 0.105	
Employment status								
Salaried (ref.)	0		0		0		0	
Self-employed	0.079		0.064		0.162		0.162	
Unemployed	− 0.111		− 0.216	**	− 0.391	***	− 0.347	**
Student	− 1.025	***	− 1.057	***	− 0.746	***	− 0.730	***
Housewife	0.609	***	0.511	***	− 0.673		− 0.636	
Inactive	− 0.118		− 0.208	***	− 0.120		− 0.055	
Other	− 0.064		− 0.163		0.095		0.168	**
Second conception								
Constant	− 7.702	***	− 7.674	***	− 8.065	***	− 8.081	***
Time since previous birth								
0–1 year (slope)	0.172	***	0.174	***	0.183	***	0.183	***
1–3 years (slope)	0.035	***	0.036	***	0.039	***	0.040	***
3–5 years (slope)	− 0.011	***	− 0.011	***	− 0.019	***	− 0.019	***
5+ years (slope)	− 0.018	***	− 0.017	***	− 0.016	***	− 0.016	***
Birth cohort								
1948–1959 (ref.)	0		0		0		0	
1960–1969	0.049		0.054		0.134	*	0.125	*
1970–1979	0.204	***	0.216	***	− 0.001		− 0.010	
1980–1999	− 0.291	***	− 0.275	**	− 0.509	***	− 0.524	***
Partnership status								
Single (ref.)	0		0		0		0	
Cohabiting	0.897	***	0.901	***	0.884	***	0.880	***
Married	1.452	***	1.469	***	1.480	***	1.473	***
Separated	0.075		0.080		0.096		0.089	
Educational level								
Low (ref.)	0		0		0		0	
Medium	− 0.218	***	− 0.238	***	− 0.106		− 0.096	
High	− 0.102		− 0.143	**	0.125		0.141	*
Origin group								
Native (ref.)	0		0		0		0	
North Africa	0.086		0.094		0.344	***	0.348	***
Sub-Saharan Africa	0.083		0.098		0.510	***	0.510	***
South East Asia	− 0.103		− 0.105		0.315	**	0.318	**
Turkey	0.175		0.197		0.454	***	0.463	***
Southern Europe	− 0.124	**	− 0.125	**	0.002		0.008	
Other Europe	− 0.076		− 0.069		0.029		0.032	
Employment status								
Salaried (ref.)	0		0		0		0	
Self-employed	− 0.032		− 0.051		0.258	**	0.259	**
Unemployed	− 0.368	***	− 0.501	***	− 0.278		− 0.239	
Student	− 0.563	***	− 0.604	***	− 0.308	*	− 0.293	*
Housewife	0.330	***	0.209	***	0.632		0.707	
Inactive	− 0.063		− 0.184	*	− 0.186		− 0.110	
Other	0.005		− 0.095		0.020		0.087	
Third conception								
Constant	− 8.071	***	− 8.026	***	− 7.904	***	− 7.928	***
Time since previous birth								
0–1 year (slope)	0.140	***	0.141	***	0.143	***	0.143	***
1–3 years (slope)	0.019	***	0.019	***	0.024	***	0.024	***
3–5 years (slope)	− 0.011	**	− 0.012	**	− 0.028	***	− 0.028	***
5+ years (slope)	− 0.022	***	− 0.022	***	− 0.017	***	− 0.017	***
Birth cohort								
1948–1959 (ref.)	0		0		0		0	
1960–1969	0.099		0.107		0.071		0.062	
1970–1979	0.156		0.163	*	− 0.020		− 0.031	
1980–1999	− 0.686	***	− 0.684	***	− 0.328		− 0.349	
Partnership status								
Single (ref.)	0		0		0		0	
Cohabiting	0.649	***	0.655	***	0.542	*	0.546	*
Married	1.062	***	1.084	***	0.893	***	0.898	***
Separated	0.377		0.376	***	0.701	**	0.705	**
Educational level								
Low (ref.)	0		0		0		0	
Medium	− 0.501	***	− 0.523	***	− 0.484	***	− 0.476	***
High	− 0.565	***	− 0.617	***	− 0.535	***	− 0.519	***
Origin group								
Native (ref.)	0		0		0		0	
North Africa	0.618	***	0.628	***	0.813	***	0.816	***
Sub− Saharan Africa	0.730	**	0.755	**	1.014	***	1.017	***
South East Asia	0.386	**	0.383	**	0.509	***	0.509	***
Turkey	0.496	***	0.526	***	0.996	***	1.004	***
Southern Europe	− 0.373	***	− 0.377	***	− 0.171		− 0.166	
Other Europe	0.311	**	0.319	**	0.370	**	0.372	**
Employment status								
Salaried (ref.)	0		0		0		0	
Self-employed	− 0.265		− 0.279		− 0.063		− 0.065	
Unemployed	0.184		0.050		0.028		0.062	
Student	0.026		− 0.021		0.013		0.030	
Housewife	0.652	***	0.520	***	0.772		0.815	
Inactive	0.122		− 0.006		0.053		0.123	
Other	0.089		− 0.010		− 0.216	*	− 0.156	
Unobserved heterogeneity								
Standard deviation of residuals								
Fertility	0.726	***	0.730	***	0.729	***	0.732	***
Employment entry	0.618	***	0.697	***	0.656	***	0.795	***
Employment exit	1.130	***	1.307	***	0.885	***	1.205	***
Correlation between residuals								
Fertility and employment entry			− 0.114	***			0.095	*
Fertility and employment exit			0.302	***			− 0.070	
Employment entry and exit			− 0.658	***			− 0.962	***
ln-L	− 96,771.47		− 96,689.48		− 76,239.96		− 76,109.02	

**p* < 0.1; ***p* < 0.05; ****p* < 0.01

^5^, 6,

**Table 6:** Impact of childbearing on employment, log-relative risks (additional specification). *Source:* Trajectories and origins, authors’ own calculations

	Women		Men	
	Model 2 multi-process		Model 2multi-process	
	RR	Sig	RR	Sig
First employment entry				
Constant	− 1.341	***	− 1.212	***
Time since in education				
0–1 year (slope)	− 0.256	***	− 0.203	***
1–3 years (slope)	0.030	***	0.027	***
3–5 years (slope)	− 0.026	***	− 0.058	***
5+ years (slope)	0.001	***	0.005	***
Birth cohort				
1948–1959 (ref.)	0		0	
1960–1969	− 0.235	***	− 0.474	***
1970–1979	− 0.194	***	− 0.447	***
1980–1999	− 0.329	***	− 0.276	***
Partnership status				
Single (ref.)	0		0	
Cohabiting	0.089	*	0.411	***
Married	− 0.209	***	0.307	***
Separated	0.317	***	0.194	*
Time since birth				
Childless (ref.)	0		0	
0–1 year after birth	− 0.746	***	0.063	
1+ years after birth	− 0.446	***	− 0.083	
Educational level				
Low (ref.)	0		0	
Medium	0.759	***	0.373	***
High	1.153	***	0.431	***
Origin group				
Native (ref.)	0		0	
North Africa	− 0.336	***	− 0.078	
Sub-Saharan Africa	− 0.337	***	− 0.230	***
South East Asia	− 0.105		0.069	
Turkey	− 0.426	***	0.503	***
Southern Europe	0.071		0.309	***
Other Europe	− 0.210	***	0.065	
Employment exits				
Constant	− 9.684	***	− 8.992	***
Time since previous employment				
0–1 year (slope)	0.319	***	0.331	***
1–3 years (slope)	− 0.024	***	− 0.039	***
3+ years (slope)	0.003	***	0.002	***
Birth cohort				
1948–1959 (ref.)	0		0	
1960–1969	0.247	**	0.351	***
1970–1979	0.642	***	0.624	***
1980–1999	1.336	***	0.886	***
Partnership status				
Single (ref.)	0		0	
Cohabiting	0.307	***	− 0.527	***
Married	0.570	***	− 0.859	***
Separated	0.444	***	− 0.081	
Time since birth				
Childless (ref.)	0		0	
0–1 year after birth	0.857	***	− 0.197	
1–3 years after birth	0.793	***	− 0.425	***
3–5 years after birth	− 0.207	**	− 0.596	***
5+ years after birth	− 0.442	***	− 0.184	
Educational level				
Low (ref.)	0		0	
Medium	− 0.544	***	− 0.459	***
High	− 1.536	***	− 1.088	***
Origin group				
Native (ref.)	0		0	
North Africa	0.249	***	0.164	*
Sub-Saharan Africa	0.069		0.153	
South East Asia	− 0.018		0.002	
Turkey	0.524	***	− 0.057	
Southern Europe	− 0.197	***	− 0.295	***
Other Europe	0.148		0.083	
Order of change				
1 (ref.)	0		0	
2	0.595	***	0.331	***
3+	0.313		0.260	**
Type of employment				
Salaried (ref.)	0		0	
Self-employed	− 0.424	***	− 0.446	***
Higher-order employment entries				
Constant	− 7.767	***	− 8.769	***
Time since out of employment				
0–1 year (slope)	0.317	***	0.498	***
1–3 years (slope)	− 0.024	***	− 0.077	***
3–5 years (slope)	− 0.016	***	− 0.019	***
5+ years (slope)	0.004	***	0.009	***
Birth cohort				
1948–1959 (ref.)	0		0	
1960–1969	0.337	***	0.108	
1970–1979	0.736	***	0.366	***
1980–1999	0.880	***	0.527	***
Partnership status				
Single (ref.)	0		0	
Cohabiting	0.081		0.334	***
Married	− 0.244	***	− 0.025	
Separated	0.031		− 0.105	
Time since birth				
Childless (ref.)	0		0	
0–1 year after birth	− 0.962	***	− 0.267	**
1–3 years after birth	− 0.895	***	− 0.205	**
3–5 years after birth	− 0.112		− 0.346	***
5+ years after birth	− 0.306	***	− 0.433	***
Educational level				
Low (ref.)	0		0	
Medium	0.625	***	0.581	***
High	1.275	***	1.161	***
Origin group				
Native (ref.)	0		0	
North Africa	− 0.119		− 0.295	**
Sub-Saharan Africa	− 0.182		− 0.402	**
South East Asia	0.025		− 0.372	**
Turkey	− 0.313	**	0.020	
Southern Europe	0.070		− 0.115	
Other Europe	− 0.044		− 0.179	
Order of change				
1 (ref.)	0		0	
2	0.404	***	0.298	***
3+	0.560	***	0.486	***
Type of out of employment				
Unemployed (ref.)	0		0	
Housewife	− 0.737	***	− 0.560	*
Other	− 0.609	***	− 0.364	***
First conception				
Constant	− 6.963	***	− 9.295	***
Age				
15–19 (slope)	0.014	***	0.040	***
20–24 (slope)	− 0.0002		0.001	
25–29 (slope)	0.004	***	0.007	***
30–34 (slope)	− 0.005	**	− 0.004	**
35+ (slope)	− 0.024	***	− 0.016	***
Birth cohort				
1948–1959 (ref.)	0		0	
1960–1969	0.146	***	− 0.018	
1970–1979	0.013		− 0.276	***
1980–1999	− 0.254	***	− 0.709	***
Partnership status				
Single (ref.)	0		0	
Cohabiting	2.253	***	2.474	***
Married	3.241	***	3.631	***
Separated	1.178	***	1.172	***
Educational level				
Low (ref.)	0		0	
Medium	− 0.287	***	− 0.082	
High	− 0.562	***	− 0.337	***
Origin group				
Native (ref.)	0		0	
North Africa	− 0.005		0.126	*
Sub-Saharan Africa	0.314	***	0.303	***
South East Asia	− 0.062		− 0.031	
Turkey	0.009		0.464	***
Southern Europe	− 0.102	**	0.101	*
Other Europe	0.025		− 0.103	
Employment Status				
Salaried (ref.)	0		0	
Self-employed	0.056		0.164	
Unemployed	− 0.230	**	− 0.375	**
Student	− 1.056	***	− 0.735	***
Housewife	0.459	***	− 0.648	
Inactive	− 0.238	***	− 0.086	
Other	− 0.170	*	0.128	
Second conception				
Constant	− 7.429	***	− 7.955	***
Time since previous birth				
0–1 year (slope)	0.174	***	0.183	***
1–3 years (slope)	0.034	***	0.039	***
3–5 years (slope)	− 0.012	***	− 0.020	***
5+ years (slope)	− 0.018	***	− 0.016	***
Birth cohort				
1948–1959 (ref.)	0		0	
1960–1969	0.051		0.135	**
1970–1979	0.166	**	− 0.008	
1980–1999	− 0.319	***	− 0.538	***
Partnership status				
Single (ref.)	0		0	
Cohabiting	0.865	***	0.864	***
Married	1.388	***	1.439	***
Separated	0.047		0.081	
Educational level				
Low (ref.)	0		0	
Medium	− 0.200	***	− 0.097	
High	− 0.048		0.162	*
Origin group				
Native (ref.)	0		0	
North Africa	0.119	*	0.354	***
Sub-Saharan Africa	0.114		0.522	***
South East Asia	− 0.096		0.332	**
Turkey	0.225	*	0.435	***
Southern Europe	− 0.110	*	0.005	
Other Europe	− 0.058		0.029	
Employment status				
Salaried (ref.)	0		0	
Self-employed	− 0.029		0.262	**
Unemployed	− 0.482	***	− 0.270	
Student	− 0.582	***	− 0.291	*
Housewife	0.186	***	0.691	
Inactive	− 0.193	*	− 0.142	
Other	− 0.075		0.048	
Third conception				
Constant	− 7.650	***	− 7.387	***
Time since previous birth				
0–1 year (slope)	0.141	***	0.143	***
1–3 years (slope)	0.019	***	0.024	***
3–5 years (slope)	− 0.012	**	− 0.028	***
5+ years (slope)	− 0.023	***	− 0.017	***
Birth cohort				
1948–1959 (ref.)	0		0	
1960–1969	0.270	**	0.067	
1970–1979	0.428	***	− 0.017	
1980–1999	− 0.319		− 0.486	
Partnership status				
Single (ref.)	0		0	
Cohabiting	− 0.194	*	0.673	**
Married	− 0.248	**	1.018	***
Separated	− 0.035		0.824	***
Educational level				
Low (ref.)	0		0	
Medium	− 0.378	***	− 0.467	***
High	− 0.104		− 0.451	***
Origin group				
Native (ref.)	0		0	
North Africa	0.474	***	0.862	***
Sub-Saharan Africa	0.641	**	1.016	***
South East Asia	0.170		0.581	***
Turkey	0.140		0.895	***
Southern Europe	− 0.392	***	− 0.155	
Other Europe	0.206		0.371	**
Employment status				
Salaried (ref.)	0		0	
Self-employed	− 0.045		− 0.054	
Unemployed	0.435	**	0.036	
Student	− 0.129		0.011	
Housewife	0.687	***	0.739	
Inactive	0.232		0.089	
Other	0.021		− 0.177	
Unobserved heterogeneity				
Standard deviation of residuals				
Fertility	0.660	***	0.698	***
Employment entry	0.661	***	0.795	***
Employment exit	1.222	***	1.205	***
Correlation between residuals				
Fertility and employment entry	− 0.194	***	0.0 57	
Fertility and employment exit	0.313	***	− 0.005	
Employment entry and exit	− 0.781	***	− 0.975	***
ln-L	− 96,381.75	− 76,095.14		

**p* < 0.1; ***p* < 0.05; ****p* < 0.01

**Table 7:** Impact of childbearing on employment by origin group, log-relative risks (Model 2, full specification). *Source:* Trajectories and Origins, authors’ own calculations

	Women		Men	
	Model 2 multi-process		Model 2 multi-process	
	RR	Sig	RR	Sig
First employment entry				
Constant	− 1.449	***	− 1.120	***
Time since in education				
0–1 year (slope)	− 0.261	***	− 0.205	***
1–3 years (slope)	0.029	***	0.027	***
3–5 years (slope)	− 0.030	***	− 0.059	***
5 + years (slope)	0.0004		0.004	***
Birth cohort				
1948–1959 (ref.)	0		0	
1960–1969	− 0.266	***	− 0.443	***
1970–1979	− 0.289	***	− 0.427	***
1980–1999	− 0.455	***	− 0.283	***
Partnership status				
Single (ref.)	0		0	
Cohabiting	0.058		0.408	***
Married	− 0.455	***	0.312	***
Separated	0.170	**	0.163	
Educational level				
Low (ref.)	0		0	
Medium	0.817	***	0.364	***
High	1.247	***	0.389	***
Employment exits				
Constant	− 9.984	***	− 8.966	***
Time since previous employment				
0–1 year (slope)	0.318	***	0.333	***
1–3 years (slope)	− 0.017	***	− 0.039	***
3+ years (slope)	0.0004		0.003	***
Birth cohort				
1948–1959 (ref.)	0		0	
1960–1969	0.329	***	0.308	***
1970–1979	0.854	***	0.586	***
1980–1999	1.559	***	0.911	***
Partnership status				
Single (ref.)	0		0	
Cohabiting	0.433	***	− 0.556	***
Married	0.670	***	− 0.867	***
Separated	0.351	***	− 0.072	
Parity × Origin group				
Childless × Natives (ref.)	0		0	
Childless × North Africa	0.260	**	− 0.091	
Childless × Sub-Saharan Africa	0.080		− 0.321	*
Childless × South East Asia	0.118		− 0.056	
Childless × Turkey	0.296		− 0.192	
Childless × Southern Europe	− 0.109		− 0.255	***
Childless × Other Europe	0.022		0.094	
1 + children × Natives	0.544	***	− 0.803	***
1 + children × North Africa	0.620	***	− 0.076	
1 + children × Sub-Saharan Africa	0.342	**	0.010	
1 + children × South East Asia	0.324	*	− 0.549	*
1 + children × Turkey	1.056	***	0.027	
1 + children × Southern Europe	0.286	***	− 0.697	***
1 + children × Other Europe	0.617	***	− 0.582	***
Educational Level				
Low (ref.)	0		0	
Medium	− 0.518	***	− 0.453	***
High	− 1.511	***	− 1.075	***
Order of change				
1 (ref.)	0		0	
2	0.270	***	0.302	***
3+	− 0.313	***	0.250	**
Type of employment				
Salaried (ref.)	0		0	
Self-employed	− 0.436	***	− 0.399	***
Higher-order employment entries				
Constant	− 8.024	***	− 8.885	***
Time since out of employment				
0–1 year (slope)	0.314	***	0.499	***
1–3 years (slope)	− 0.020	***	− 0.077	***
3–5 years (slope)	− 0.013	***	− 0.018	***
5 + years (slope)	0.005	***	0.009	***
Birth cohort				
1948–1959 (ref.)	0		0	
1960–1969	0.297	***	0.151	**
1970–1979	0.645	***	0.419	***
1980–1999	0.806	***	0.585	***
Partnership status				
Single (ref.)	0		0	
Cohabiting	0.006		0.336	***
Married	− 0.313	***	− 0.019	
Separated	0.032		− 0.121	
Parity × Origin group				
Childless × Natives (ref.)	0		0	
Childless × North Africa	0.197		− 0.168	
Childless × Sub-Saharan Africa	− 0.123		− 0.275	
Childless × South East Asia	0.314	*	− 0.338	**
Childless × Turkey	0.193		− 0.178	
Childless × Southern Europe	0.333	***	− 0.160	
Childless × Other Europe	0.242		− 0.049	
1 + children × Natives	− 0.466		− 0.419	
1 + children × North Africa	− 0.343	***	− 0.583	***
1 + children × Sub-Saharan Africa	− 0.022		− 0.376	
1 + children × South East Asia	− 0.198		− 0.517	*
1 + children × Turkey	− 0.640	***	− 0.126	
1 + children × Southern Europe	− 0.206		− 0.428	***
1 + children × Other Europe	− 0.251		− 0.635	***
Educational level				
Low (ref.)	0		0	
Medium	0.639	***	0.582	***
High	1.316	***	1.142	***
Order of change				
1 (ref.)	0		0	
2	0.424	***	0.299	***
3 +	0.615	***	0.474	***
Type of out of employment				
Unemployed (ref.)	0		0	
Housewife	− 0.779	***	− 0.525	*
Other	− 0.610	***	− 0.360	***
First conception				
Constant	− 7.013	***	− 10.253	***
Age				
15–19 (slope)	0.015	***	0.101	
20–24 (slope)	0.001		0.058	***
25–29 (slope)	0.005	***	0.022	***
30–34 (slope)	− 0.004	**	0.008	***
35+ (slope)	− 0.024	***	− 0.003	***
Birth cohort				
1948–1959 (ref.)	0		0	
1960–1969	0.148	***	0.044	
1970–1979	0.012		− 0.185	***
1980–1999	− 0.241	***	− 0.728	***
Partnership status				
Single (ref.)	0		0	
Cohabiting	2.263	***	2.473	***
Married	3.269	***	3.646	***
Separated	1.176	***	1.215	***
Educational level				
Low (ref.)	0		0	
Medium	− 0.325	***	− 0.099	*
High	− 0.633	***	− 0.300	***
Origin group				
Native (ref.)	0		0	
North Africa	− 0.042		0.152	**
Sub-Saharan Africa	0.299	***	0.345	***
South East Asia	− 0.074		− 0.009	
Turkey	− 0.018		0.449	***
Southern Europe	− 0.102	*	0.094	*
Other Europe	0.009		− 0.107	
Employment status				
Salaried (ref.)	0		0	
Self-employed	0.066		0.203	**
Unemployed	− 0.266	***	− 0.437	***
Student	− 1.081	***	− 0.781	***
Housewife	0.432	***	− 0.738	
Inactive	− 0.297	***	− 0.128	
Other	− 0.224	**	0.152	*
Second conception				
Constant	− 7.653	***	− 8.039	***
Time since previous birth				
0–1 year (slope)	0.174	***	0.183	***
1–3 years (slope)	0.035	***	0.039	***
3–5 years (slope)	− 0.011	***	− 0.020	***
5+ years (slope)	− 0.017	***	− 0.016	***
Birth cohort				
1948–1959 (ref.)	0		0	
1960–1969	0.060		0.142	**
1970–1979	0.229	***	0.025	
1980–1999	− 0.259	**	− 0.490	***
Partnership status				
Single (ref.)	0		0	
Cohabiting	0.906	***	0.860	***
Married	1.484	***	1.449	***
Separated	0.090		0.074	
Educational level				
Low (ref.)	0		0	
Medium	− 0.254	***	− 0.102	
High	− 0.172	**	0.128	
Origin group				
Native (ref.)	0		0	
North Africa	0.067		0.343	***
Sub-Saharan Africa	0.065		0.505	***
South East Asia	− 0.120		0.309	**
Turkey	0.166		0.446	***
Southern Europe	− 0.121	**	0.002	
Other Europe	− 0.082		0.032	
Employment status				
Salaried (ref.)	0		0	
Self-employed	− 0.057		0.252	**
Unemployed	− 0.538	***	− 0.282	
Student	− 0.633	***	− 0.302	*
Housewife	0.164	***	0.665	
Inactive	− 0.270	***	− 0.163	
Other	− 0.137		0.033	
Third conception				
Constant	− 8.003	***	− 7.874	***
Time since previous birth				
0–1 year (slope)	0.141	***	0.143	***
1–3 years (slope)	0.019	***	0.024	***
3–5 years (slope)	− 0.012	**	− 0.028	***
5 + years (slope)	− 0.023	***	− 0.017	***
Birth Cohort				
1948–1959 (ref.)	0		0	
1960–1969	0.110		0.083	
1970–1979	0.175	*	0.015	
1980–1999	− 0.676	***	− 0.308	
Partnership status				
Single (ref.)	0		0	
Cohabiting	0.655	***	0.518	
Married	1.093	***	0.865	***
Separated	0.381		0.679	**
Educational level				
Low (ref.)	0		0	
Medium	− 0.533	***	− 0.480	***
High	− 0.636	***	− 0.533	***
Origin group				
Native (ref.)	0		0	
North Africa	0.597	***	0.810	***
Sub-Saharan Africa	0.715	***	1.011	***
South East Asia	0.369	**	0.501	***
Turkey	0.509	***	0.981	***
Southern Europe	− 0.373	***	− 0.174	
Other Europe	0.305	**	0.368	**
Employment status				
Salaried (ref.)	0		0	
Self-employed	− 0.278		− 0.074	
Unemployed	0.015		0.023	
Student	− 0.038		0.021	
Housewife	0.481	***	0.790	
Inactive	− 0.084		0.065	
Other	− 0.047		− 0.201	*
Unobserved heterogeneity				
Standard deviation of residuals				
Fertility	0.731	***	0.709	***
Employment entry	0.681	***	0.796	***
Employment exit	1.325	***	1.249	***
Correlation between residuals				
Fertility and employment entry	− 0.290	***	0.044	
Fertility and employment exit	0.312	***	0.016	
Employment entry and exit	− 0.665	***	− 0.937	***
ln-L	− 96,807.59		− 76,225.16	

^*^*p* < 0.1; ***p* < 0.05; ****p* < 0.01

^7^, 8, and

**Table 8:** Impact of childbearing on employment, log-relative risks (Model 1, with 10 integration points). *Source:* Trajectories and Origins, authors’ own calculations

	Women				Men			
	Model 1a single-process		Model 1b multi-process		Model 1a single-process		Model 1b multi-process	
	RR	Sig	RR	Sig	RR	Sig	RR	Sig
First employment entry								
Constant	− 1.294	***	− 1.364	***	− 1.105	***	− 1.219	***
Time since in education								
0–1 year (slope)	− 0.260	***	− 0.251	***	− 0.216	***	− 0.201	***
1–3 years (slope)	0.032	***	0.031	***	0.025	***	0.027	***
3–5 years (slope)	− 0.026	***	− 0.024	***	− 0.061	***	− 0.058	***
5+ years (slope)	0.001		0.001		0.004	***	0.005	***
Birth cohort								
1948–1959 (ref.)	0		0		0		0	
1960–1969	− 0.246	***	− 0.238	***	− 0.464	***	− 0.475	***
1970–1979	− 0.218	***	− 0.199	***	− 0.437	***	− 0.443	***
1980–1999	− 0.358	***	− 0.340	***	− 0.300	***	− 0.274	***
Partnership status								
Single (ref.)	0		0		0		0	
Cohabiting	0.094	**	0.079		0.390	***	0 .411	***
Married	− 0.189	***	− 0.225	***	0.283	***	0.303	***
Separated	0.350	***	0.339	***	0.136		0.190	*
Parity								
Childless (ref.)	0		0		0		0	
With children	− 0.642	***	− 0.630	***	− 0.036		− 0.050	
Educational level								
Low (ref.)	0		0		0		0	
Medium	0.748	***	0.775	***	0.358	***	0.375	***
High	1.140	***	1.180	***	0.390	***	0.435	***
Origin group								
Native (ref.)	0		0		0		0	
North Africa	− 0.343	***	− 0.339	***	− 0.094	*	− 0.080	
Sub-Saharan Africa	− 0.328	***	− 0.332	***	− 0.241	***	− 0.232	***
South East Asia	− 0.109		− 0.105		0.050		0.069	
Turkey	− 0.424	***	− 0.429	***	0.445	***	0.497	***
Southern Europe	0.071		0.071		0.283	***	0.310	***
Other Europe	− 0.205	***	− 0.211	***	0.065		0.065	
Employment exits								
Constant	− 10.073	***	− 9.921	***	− 8.948	***	− 8.994	***
Time since previous employment								
0–1 year (slope)	0.316	***	0.318	***	0.329	***	0.332	***
1–3 years (slope)	− 0.020	***	− 0.018	***	− 0.044	***	− 0.039	***
3+ years (slope)	− 0.001	*	0.001		0.001	***	0.002	***
Birth cohort								
1948–1959 (ref.)	0		0		0		0	
1960–1969	0.259	***	0.296	***	0.176	*	0.329	***
1970–1979	0.748	***	0.794	***	0.476	***	0.602	***
1980–1999	1.377	***	1.485	***	0.612	***	0.874	***
Partnership status								
Single (ref.)	0		0		0		0	
Cohabiting	0.359	***	0.403	***	− 0.509	***	− 0.536	***
Married	0.499	***	0.644	***	− 0.874	***	− 0.904	***
Separated	0.288	***	0.325	***	− 0.004		− 0.091	
Parity								
Childless (ref.)	0		0		0		0	
1 child	0.674	***	0.526	***	− 0.349	***	− 0.377	***
2 children	0.732	***	0.442	***	− 0.246	**	− 0.313	**
3+ children	0.882	***	0.365	***	− 0.046		0.007	
Educational level								
Low (ref.)	0		0		0		0	
Medium	− 0.304	***	− 0.509	***	− 0.331	***	− 0.456	***
High	− 1.228	***	− 1.491	***	− 0.903	***	− 1.097	***
Origin group								
Native (ref.)	0		0		0		0	
North Africa	0.226	***	0.266	***	0.196	**	0.158	*
Sub-Saharan Africa	− 0.044		0.075		0.133		0.123	
South East Asia	− 0.014		− 0.011		0.052		− 0.002	
Turkey	0.465	***	0.569	***	0.028		− 0.084	
Southern Europe	− 0.159	**	− 0.207	***	− 0.195	**	− 0.296	***
Other Europe	0.102		0.147		0.160		0.083	
Order of change								
1 (ref.)	0		0		0		0	
2	0.066		0.293	***	0.188	***	0.306	***
3 +	− 0.676	***	− 0.270	**	− 0.031		0.224	**
Type of employment								
Salaried (ref.)	0		0		0		0	
Self-employed	− 0.399	***	− 0.432	***	− 0.439	***	− 0.445	***
Higher-order employment entries								
Constant	− 7.763	***	− 7.714	***	− 8.869	***	− 8.799	***
Time since out of employment								
0–1 year (slope)	0.312	***	0.315	***	0.488	***	0.498	***
1–3 years (slope)	− 0.020	***	− 0.019	***	− 0.081	***	− 0.077	***
3–5 years (slope)	− 0.012	***	− 0.0 11	***	− 0.018	***	− 0.018	***
5+ years (slope)	0.005	***	0.005	***	0.008	***	0.009	***
Birth cohort								
1948–1959 (ref.)	0		0		0		0	
1960–1969	0.290	***	0.283	***	0.166	**	0.132	*
1970–1979	0.617	***	0.613	***	0.443	***	0.396	***
1980–1999	0.768	***	0.743	***	0.561	***	0.554	***
Partnership status								
Single (ref.)	0		0		0		0	
Cohabiting	0.031		0.033		0.227	***	0.331	***
Married	− 0.222	***	− 0.228	***	− 0.161	**	− 0.017	
Separated	0.079		0.086		− 0.240	***	− 0.118	
Parity								
Childless (ref.)	0		0		0		0	
1 child	− 0.520	***	− 0.504	***	− 0.234	***	− 0.221	***
2 children	− 0.721	***	− 0.708	***	− 0.449	***	− 0.428	***
3+ children	− 0.972	***	− 0.983	***	− 0.387	***	− 0.430	***
Educational level								
Low (ref.)	0		0		0		0	
Medium	0.568	***	0.636	***	0.517	***	0.578	***
High	1.182	***	1.327	***	1.097	***	1.163	***
Origin group								
Native (ref.)	0		0		0		0	
North Africa	− 0.114		− 0.120		− 0.259	**	− 0.286	**
Sub-Saharan Africa	− 0.151		− 0.185		− 0.330	**	− 0.375	**
South East Asia	− 0.002		0.017		− 0.372	**	− 0.361	**
Turkey	− 0.247		− 0.309	*	− 0.046		0.021	
Southern Europe	0.048		0.075		− 0.147		− 0.111	
Other Europe	− 0.046		− 0.058		− 0.165		− 0.182	
Order of change								
1 (ref.)	0		0		0		0	
2	0.403	***	0.466	***	0.248	***	0.289	***
3+	0.527	***	0.696	***	0.295	***	0.482	***
Type of out of employment								
Unemployed (ref.)	0		0		0		0	
Housewife	− 0.732	***	− 0.764	***	− 0.523	*	− 0.540	*
Other	− 0.555	***	− 0.624	***	− 0.329	***	− 0.356	***
First conception								
Constant	− 7.037	***	− 7.025	***	− 9.308	***	− 9.316	***
Age								
15–19 (slope)	0.014	***	0.015	***	0.040	***	0.039	***
20–24 (slope)	0.001		0.001		0.001		0.001	
25–29 (slope)	0.005	***	0.005	***	0.007	***	0.008	***
30–34 (slope)	− 0.004	**	− 0.004	**	− 0.003	**	− 0.003	**
35+ (slope)	− 0.024	***	− 0.024	***	− 0.016	***	− 0.016	***
Birth cohort								
1948–1959 (ref.)	0		0		0		0	
1960–1969	0.124	**	0.133	**	− 0.018		− 0.026	
1970–1979	− 0.021		− 0.008		− 0.280	***	− 0.288	***
1980–1999	− 0.288	***	− 0.268	***	− 0.712	***	− 0.723	***
Partnership status								
Single (ref.)	0		0		0		0	
Cohabiting	2.249	***	2.258	***	2.480	***	2.478	***
Married	3.250	***	3.260	***	3.644	***	3.643	***
Separated	1.166	***	1.171		1.174	***	1.175	***
Educational level								
Low (ref.)	0		0		0		0	
Medium	− 0.301	***	− 0.313	***	− 0.092		− 0.086	
High	− 0.592	***	− 0.613	***	− 0.362	***	− 0.351	***
Origin group								
Native (ref.)	0		0		0		0	
North Africa	− 0.029		− 0.018		0.116	*	0.119	*
Sub-Saharan Africa	0.320	***	0.321	***	0.311	***	0.310	***
South East Asia	− 0.073		− 0.069		− 0.040		− 0.036	
Turkey	0.001		0.011		0.471	***	0.483	***
Southern Europe	− 0.099	*	− 0.103	**	0.096	*	0.100	*
Other Europe	0.017		0.022		− 0.109		− 0.107	
Employment status								
Salaried (ref.)	0		0		0		0	
Self-employed	0.079		0.063		0.161		0.161	
Unemployed	− 0.111		− 0.214	**	− 0.391	***	− 0.351	**
Student	− 1.025	***	− 1.056	***	− 0.746	***	− 0.732	***
Housewife	0.611	***	0.515	***	− 0.674		− 0.641	
Inactive	− 0.118		− 0.205	***	− 0.119		− 0.061	
Other	− 0.064		− 0.162		0.095		0.161	*
Second conception								
Constant	− 7.699	***	− 7.674	***	− 8.064	***	− 8.078	***
Time Since Previous Birth								
0–1 year (slope)	0.172	***	0.174	***	0.183	***	0.183	***
1–3 years (slope)	0.035	***	0.036	***	0.039	***	0.040	***
3–5 years (slope)	− 0.011	***	− 0.011	***	− 0.019	***	− 0.019	***
5+ years (slope)	− 0.018	***	− 0.017	***	− 0.016	***	− 0.016	***
Birth cohort								
1948–1959 (ref.)	0		0		0		0	
1960–1969	0.048		0.053		0.133	*	0.125	*
1970–1979	0.203	***	0.214	***	− 0.002		− 0.010	
1980–1999	− 0.292	***	− 0.277	**	− 0.509	***	− 0.524	***
Partnership status								
Single (ref.)	0		0		0		0	
Cohabiting	0.897	***	0.901	***	0.883	***	0.880	***
Married	1.450	***	1.467	***	1.479	***	1.474	***
Separated	0.074		0.080		0.096		0.091	
Educational Level								
Low (ref.)	0		0		0		0	
Medium	− 0.218	***	− 0.237	***	− 0.105		− 0.096	
High	− 0.103		v0.143	**	0.127		0.141	*
Origin group								
Native (ref.)	0		0		0		0	
North Africa	0.086		0.094		0.344	***	0.347	***
Sub-Saharan Africa	0.081		0.096		0.517	***	0.516	***
South East Asia	− 0.101		− 0.103		0.317	**	0.320	**
Turkey	0.177		0.201		0.453	***	0.460	***
Southern Europe	− 0.123	**	− 0.123	**	0.001		0.006	
Other Europe	− 0.076		− 0.068		0.028		0.030	
Employment status								
Salaried (ref.)	0		0		0		0	
Self-employed	− 0.027		− 0.045		0.257	**	0.257	**
Unemployed	− 0.368	***	− 0.500	***	− 0.278		− 0.243	
Student	− 0.563	***	− 0.604	***	− 0.309	*	− 0.294	*
Housewife	0.331	***	0.211	***	0.623		0.680	
Inactive	− 0.063		− 0.181	*	− 0.183		− 0.115	
Other	0.005		− 0.093		0.019		0.080	
Third conception								
Constant	− 8.069	***	− 8.026	***	− 7.900	***	− 7.923	***
Time since previous birth								
0–1 year (slope)	0.140	***	0.141	***	0.143	***	0.143	***
1–3 years (slope)	0.019	***	0.019	***	0.024	***	0.024	***
3–5 years (slope)	− 0.011	**	− 0.012	**	− 0.028	***	− 0.028	***
5+ years (slope)	− 0.022	***	− 0.022	***	− 0.017	***	− 0.017	***
Birth cohort								
1948–1959 (ref.)	0		0		0		0	
1960–1969	0.099		0.106		0.069		0.062	
1970–1979	0.154		0.162		− 0.022		− 0.031	
1980–1999	− 0.685	***	− 0.684	***	− 0.329		− 0.348	
Partnership status								
Single (ref.)	0		0		0		0	
Cohabiting	0.650	***	0.656	***	0.540	*	0.546	*
Married	1.061	***	1.082	***	0.892	***	0.898	***
Separated	0.377		0.378		0.700	**	0.705	**
Educational level								
Low (ref.)	0		0		0		0	
Medium	− 0.500	***	− 0.523	***	− 0.484	***	− 0.476	***
High	− 0.565	***	− 0.617	***	− 0.535	***	− 0.519	***
Origin group								
Native (ref.)	0		0		0		0	
North Africa	0.618	***	0.628	***	0.812	***	0.814	***
Sub-Saharan Africa	0.730	***	0.755	***	1.019	***	1.023	***
South East Asia	0.386	**	0.384	**	0.509	***	0.510	***
Turkey	0.495	***	0.527	***	0.994	***	1.000	***
Southern Europe	− 0.372	***	− 0.375	***	− 0.173		− 0.168	
Other Europe	0.312	**	0.320	**	0.369	**	0.370	**
Employment status								
Salaried (ref.)	0		0		0		0	
Self-employed	− 0.263		− 0.277		− 0.063		− 0.065	
Unemployed	0.184		0.052		0.027		0.056	
Student	0.025		− 0.022		0.009		0.026	
Housewife	0.652	***	0.522	***	0.771		0.814	
Inactive	0.120		− 0.003		0.053		0.116	
Other	0.088		− 0.010		− 0.217	*	− 0.162	
Unobserved heterogeneity								
Standard deviation of residuals								
Fertility	0.725	***	0.730	***	0.729	***	0.731	***
Employment entry	0.619	***	0.700	***	0.659	***	0.809	***
Employment exit	1.200	***	1.305	***	0.873	***	1.222	***
Correlation between residuals								
Fertility and employment entry			− 0.107	**			0.085	*
Fertility and employment exit			0.301	***			− 0.065	
Employment entry and exit			− 0.656	***			− 0.940	***
ln-L	− 96,770.46		− 96,689.12		− 76,239.74		− 76,107.76	

**p* < 0.1; ***p* < 0.05; ****p* < 0.01

**Table 9:** Impact of childbearing on employment by origin group, log-relative risks (Model 2 with 10 integration points). *Source:* Trajectories and Origins, authors’ own calculations

	Women		Men	
	Model 2 multiprocess		Model 2 multiprocess	
	RR	Sig	RR	Sig
First employment entry				
Constant	− 1.451	***	− 1.126	***
Time since in education				
0–1 year (slope)	− 0.260	***	− 0.203	***
1–3 years (slope)	0.029	***	0.027	***
3–5 years (slope)	− 0.030	***	− 0.058	***
5+ years (slope)	0.0004		0.004	***
Birth cohort				
1948–1959 (ref.)	0		0	
1960–1969	− 0.264	***	− 0.449	***
1970–1979	− 0.288	***	− 0.426	***
1980–1999	− 0.454	***	− 0.284	***
Partnership status				
Single (ref.)	0		0	
Cohabiting	0.058		0.406	***
Married	− 0.456	***	0.305	***
Separated	0.170	**	0.167	
Educational level				
Low (ref.)	0		0	
Medium	0.818	***	0.366	***
High	1.247	***	0.395	***
Employment exits				
Constant	− 9.982	***	− 8.968	***
Time since previous employment				
0–1 year (slope)	0.318	***	0.333	***
1–3 years (slope)	− 0.017	***	− 0.039	***
3+ years (slope)	0.0004		0.003	***
Birth cohort				
1948–1959 (ref.)	0		0	
1960–1969	0.328	***	0.305	***
1970–1979	0.852	***	0.576	***
1980–1999	1.557	***	0.905	***
Partnership status				
Single (ref.)	0		0	
Self-employed	− 0.277		− 0.075	
Unemployed	0.016		0.018	
Student	− 0.040		0.017	
Housewife	0.482	***	0.789	
Inactive	− 0.083		0.059	
Other	− 0.048		− 0.205	*
Unobserved heterogeneity				
Standard deviation of residuals				
Fertility	0.731	***	0.710	***
Employment entry	0.682	***	0.815	***
Employment exit	1.323	***	1.265	***
Correlation between residuals				
Fertility and employment entry	− 0.287	***	0.037	
Fertility and employment exit	0.312	***	0.019	
Employment entry and exit	− 0.666	***	− 0.922	***
ln-L	− 96807.89		-76222.99	

**p* < 0.1; ***p* < 0.05; ****p* < 0.01

^9^.

## Footnotes

1 This has been done in previous existing studies in order to avoid introducing more sources of endogeneity (Miettinen & Jalovaara, 2020 among others).

2 We distinguish between first employment entry and higher-order employment entries due to the fact that the first school-to-work transition is likely to differ compared to other later employment entries.

3 The first, second, and third birth are modelled as separate processes due to the fact that we expect socio-demographic characteristics to play a different role on the propensity to experience each event.

4 We report the results of an additional model in the Appendix where we also look at the effect of time since childbirth on the employment changes of individuals.

5 The results are reported in the Appendix Tables 8 and 9.

6 This is due to the fact that we do not observe many individuals having children at the time of the first school-to-work transition.

7 Individuals from Southern European origin have been distinguished from individuals of other European origin due to the fact that they constitute a sizeable origin group in the French immigrant population. We did not differentiate further between individuals of Western and Eastern European origins due to limited sample sizes.
